# Methodological choices affect cancer incidence rates: a cohort study

**DOI:** 10.1186/s12963-017-0120-x

**Published:** 2017-01-19

**Authors:** Hannah L. Brooke, Mats Talbäck, Maria Feychting, Rickard Ljung

**Affiliations:** Unit of Epidemiology, Institute of Environmental Medicine, Karolinska Institutet, 171 77, Stockholm, PO Box 210, Sweden

**Keywords:** Incidence rate, Cancer, Methods, Study population, Standardized incidence ratio

## Abstract

**Background:**

Incidence rates are fundamental to epidemiology, but their magnitude and interpretation depend on methodological choices. We aimed to examine the extent to which the definition of the study population affects cancer incidence rates.

**Methods:**

All primary cancer diagnoses in Sweden between 1958 and 2010 were identified from the national Cancer Register. Age-standardized and age-specific incidence rates of 29 cancer subtypes between 2000 and 2010 were calculated using four definitions of the study population: persons resident in Sweden 1) based on general population statistics; 2) with no previous subtype-specific cancer diagnosis; 3) with no previous cancer diagnosis except non-melanoma skin cancer; and 4) with no previous cancer diagnosis of any type. We calculated absolute and relative differences between methods.

**Results:**

Age-standardized incidence rates calculated using general population statistics ranged from 6% lower (prostate cancer, incidence rate difference: -13.5/100,000 person-years) to 8% higher (breast cancer in women, incidence rate difference: 10.5/100,000 person-years) than incidence rates based on individuals with no previous subtype-specific cancer diagnosis. Age-standardized incidence rates in persons with no previous cancer of any type were up to 10% lower (bladder cancer in women) than rates in those with no previous subtype-specific cancer diagnosis; however, absolute differences were <5/100,000 person-years for all cancer subtypes.

**Conclusions:**

For some cancer subtypes incidence rates vary depending on the definition of the study population. For these subtypes, standardized incidence ratios calculated using general population statistics could be misleading. Moreover, etiological arguments should be used to inform methodological choices during study design.

**Electronic supplementary material:**

The online version of this article (doi:10.1186/s12963-017-0120-x) contains supplementary material, which is available to authorized users.

## Background

Incidence rates are fundamental to descriptive epidemiology for quantifying disease occurrence in populations, and to analytical epidemiology for comparing disease occurrence according to exposure status [1]. They are calculated simply as the number of new cases of disease per unit of person-time at risk of becoming a case. However, calculating incidence rates for cancer is more complex, since one individual may have multiple primary cancer diagnoses over time. How to handle multiple cancers is a common design issue to be considered in any cohort study.

Cancer registries often calculate incidence rates based on aggregate general population statistics, i.e., the total number of new primary tumors recorded each year divided by the mean population that year, regardless of previous cancer diagnoses and exact person-time accumulated [2, 3]. As such, prevalent cases are included in both the numerator and the denominator. When individual-level data are available, the study population can be defined more precisely, usually in one of three ways: 1) persons with no previous diagnosis of the cancer subtype of interest, e.g., [4]; 2) persons with no previous diagnosis of any cancer subtype except non-melanoma skin cancer, e.g., [5]; and 3) persons with no previous diagnosis of any cancer subtype, e.g., [6]. Moreover, individual-level data enable person-time to be measured exactly. Variation in the precision of the numerator and the denominator may cause incidence rates based on aggregate population data to deviate from incidence rates based on individual-level data. However, the magnitude and direction of deviation between these methods is unclear. It is essential to evaluate these differences as standardized incidence ratios, calculated to examine the effect of an exposure or intervention in a subpopulation with individual-level data, often depend on aggregate general population statistics to estimate the expected number of cases.

In studies of cancer incidence with individual-level data, the choice of study population is important and may influence the estimated incidence rate. For example, if a cancer diagnosis is associated with higher incidence of a second cancer subtype, then the incidence rate of the second cancer subtype will be higher if persons with a previous cancer diagnosis are included in the calculation than if they were excluded. However, the most appropriate definition for the study population may not be clear and depends on the research question at hand. For descriptive purposes it would be prudent to include all individuals with a new primary cancer diagnosis regardless of previous cancer diagnoses. However, for analytical epidemiology, whether individuals with a previous cancer diagnosis should be included in the study population depends on whether the previous cancer is considered to be a confounder (i.e., associated with the exposure and the second cancer of interest). For example, a previous cancer diagnosis may lead to changes in lifestyle or behavior, while the treatment of a previous cancer can affect future cancer risk. Cancer diagnostics and treatments continue to improve, so the number of cancer survivors at risk of a new cancer diagnosis continues to increase [7]. It is therefore important to examine how the definition of the study population influences estimates of cancer incidence rates, particularly as variation in the methods used to calculate cancer incidence rate may reduce comparability between studies. Although the extent to which such methodological choices influence the overall incidence rate may have been examined within cancer registries, to our knowledge this has not previously been quantified in peer-reviewed scientific literature.

We aimed to evaluate the magnitude and direction of deviation between incidence rates calculated from aggregate general population statistics and individual-level data. We further aimed to assess the extent of differences in cancer incidence rates calculated using three common definitions of the study population in individual-level data. Although we focus on cancer incidence rates, the principles of this paper may also be relevant for other disease outcomes.

## Methods

### Study design

We conducted a population-based open cohort study of all individuals officially resident in Sweden between January 1, 2000, and December 31, 2010. We used the Total Population Register to identify the cohort and to ascertain age and sex [8]. The cohort was linked to the Cancer Register and the Cause of Death Register using the unique personal identity number assigned to each individual registered in Sweden [9]. All primary malignant cancer diagnoses between January 1, 1958, and December 31, 2010, were identified from the Cancer Register. The Cancer register has an estimated completeness of at least 96%; however, it is not considered complete before 1960 [10]. Aggregate general population statistics on the mean annual population between 2000 and 2010 were retrieved from Statistics Sweden. In the individual-level analyses, follow-up began on January 1, 2000, and participants were censored on 1) emigration before December 31, 2010, 2) death before December 31, 2010, or 3) end of study period, i.e., December 31, 2010. Ethical approval for the study was granted by the Regional Ethical Review Board, Stockholm, Sweden (2011/634-31/4).

### Outcomes

We categorized cancer into 29 subtype groups in accordance with the cancer dictionary used in the World Health Organization (WHO) cancer mortality database [11]. Coding was based on the International Statistical Classification of Diseases and Related Health Problems, Seventh Revision (ICD-7), as this was available for the whole period 1958–2010. The 29 cancer categories (and corresponding ICD-10 codes) were lip, oral cavity and pharynx (C00-C14); nasopharynx (C11); esophagus (C15); stomach (C16); intestine (C17-C21); colon (C18); colon, rectum, and anus (C18-C21); rectum and anus (C19-C21); liver (specified as primary) (C22); gallbladder (C23-C24); pancreas (C25); larynx (C32); lung (including trachea and bronchus) (C33-C34); melanoma of skin (C43); breast (C50, female only); uterus (C53-C55); cervix uteri (C53); corpus uteri (C54); ovary (C56); prostate (C61); testis (C62); kidney (C64); bladder (C67); brain and central nervous system (C70-C72); thyroid (C73); Hodgkin lymphoma (C81); non-Hodgkin lymphoma (C82-C86, C96); multiple myeloma (C88 + C90); and leukemia (C91-C95) [11]. The four WHO categories, intestines (C17-C21), colon (C18), colon, rectum, and anus (C18-C21), and rectum and anus (C19-C21), are overlapping groups. All groups were included for consistency with the classification system. However, when discussed as a whole, these groups will be referred to herein as colorectal cancer. Although there were cases of male breast cancer, these were not presented since there were very low numbers.

### Statistical analysis

We calculated crude, age-standardized, and age-group-specific (age groups: 0–24, 25–44, 45–64, 65–84, 85+ years) incidence rates for each cancer subtype, using four different methods (for further explanation see Table 1):

**Table 1:** Four definitions of the study population applied to hypothetical data from seven patients

	Year of diagnosis			Number of breast cancer diagnoses counted for each patient for each different definition of the study population			
	Prior to study period 1958–1999	Study period 2000–2010					
Patient	1987	2002	2009	Aggr^a^	Subtype^b^	xNMSC^c^	First ever^d^
A^e^	Breast	-	-	0	0	0	0
B^f^	-	Breast	-	1	1	1	1
C^g^	NMSC	Breast	-	1	1	1	0
D^h^	Colon	Breast	-	1	1	0	0
E^i^	Breast	Breast	-	1	0	0	0
F^j^	-	Breast	Colon	1	1	1	1
G^k^	-	Breast	Breast	2	1	1	1

Cancer diagnoses prior to (1958–1999) and during (2000–2010) the study period, and number of breast cancer (BC) diagnoses counted using each definition of the study population (SP)

^a^Aggr, SP based on aggregate general population statistics

^b^Subtype, SP excluding individuals with a previous diagnosis of the cancer subtype of interest

^c^xNMSC, SP excluding individuals with any previous cancer diagnosis, except if the previous cancer was non-melanoma skin cancer

^d^First ever, SP excluding individuals with any previous cancer diagnosis

^e^Patient A: excluded from all methods as BC in 1987 was prior to the study period

^f^Patient B: counted in all methods as there was no previous cancer diagnosis before BC in 2002

^g^Patient C: BC in 2002 is counted in Aggr, Subtype and xNMSC, but not in First ever as the first ever cancer diagnosis was a non-melanoma skin cancer (NMSC) in 1987

^h^Patient D: BC in 2002 is counted once in Aggr and once in Subtype, as it is the first subtype specific cancer. However, due to a previous colon cancer diagnosis in 1987 BC in 2002 is not counted in xNMSC or First ever

^I^Patient E: BC in 2002 is counted once in Aggr. However, the patient is excluded from all other methods as the first BC occurred in 1987, which is not within the study period

^j^Patient F: BC in 2002 is counted once in all methods (similar to patient B). The diagnosis of colon cancer in 2009 does not influence the incidence of BC

^k^Patient G: BC in 2002 and 2009 are counted as two cancers in Aggr, as there are two records of new primary tumours in the study period. However, only the BC in 2002 is counted in the other methods as the first diagnosis

1. Incidence rates calculated from aggregate general population statistics, herein referred to as aggregate population incidence rates. All new primary malignant tumors recorded in the Cancer Register during the study period were included. The person-time at risk was estimated as the mean population each year, summed over the study period. This replicates the method used by cancer registries to calculate incidence rates [2, 3].
2. Incidence rates calculated from individual-level data with the study population defined as persons with no previous subtype-specific cancer diagnosis, i.e., excluding individuals with a previous diagnosis of the cancer subtype of interest, herein referred to as subtype-specific incidence rates.
3. Incidence rates calculated from individual data with the study population defined as persons with no previous cancer diagnosis of any type except non-melanoma skin cancer, i.e., excluding individuals with any previous cancer diagnosis, except if the previous cancer was non-melanoma skin cancer, herein referred to as first cancer except non-melanoma skin cancer incidence rates.
4. Incidence rates calculated from individual data with the study population defined as persons with no previous cancer diagnosis of any type, i.e., excluding individuals with any previous cancer diagnosis, herein referred to as first-ever cancer incidence rates.

Age-standardized incidence rates were calculated as described by the International Agency for Research on Cancer [3]. Incidence rates standardized to the world standard population suggested by Segi 1960 and revised by Doll et al., 1966 are presented in the results [3]. In addition, incidence rates standardized to the Swedish population in 2000 are provided in Additional File 1: Table S1 [2].

We calculated incidence rate differences (IRD) and incidence rate ratios (IRR) for each different method of calculating incidence rates. We used subtype-specific incidence rates as the reference rates. As such, there are six comparisons (three IRD and three IRR) for each cancer subtype: 1) aggregate population incidence rates (any cancer diagnosis during the study period) vs. subtype-specific incidence rates (first cancer of that subtype); 2) first cancer except non-melanoma skin cancer incidence rates (first cancer of any type, except non-melanoma skin cancer) vs. subtype-specific incidence rates (first cancer of that subtype); and 3) first-ever cancer incidence rates (first cancer of any type) vs. subtype-specific incidence rates (first cancer of that subtype).

## Results

### Cohort description

In total 10,515,591 individuals (49.7% males) were included in the cohort. Based on aggregate population data, a total of 99,799,233 years of person-time was accumulated, of which 29.8%, 26.9%, 25.9%, 15%, and 2.5% was in the age groups 0–24, 25–44, 45–64, 65–84, and 85+ years, respectively. During the study period 476,719 new primary tumors, in mutually exclusive cancer subtypes, were reported to the Cancer Registry. Based on individual-level data, 459,174 of these tumors were diagnosed in persons with no previous subtype-specific cancer diagnosis, 410,428 were diagnosed in persons with no previous cancer diagnosis of any type except non-melanoma skin cancer, and 406,633 were diagnosed in persons with no previous cancer diagnosis of any type.

### Aggregate population incidence rates compared to subtype-specific incidence rates

**Fig. 1:**
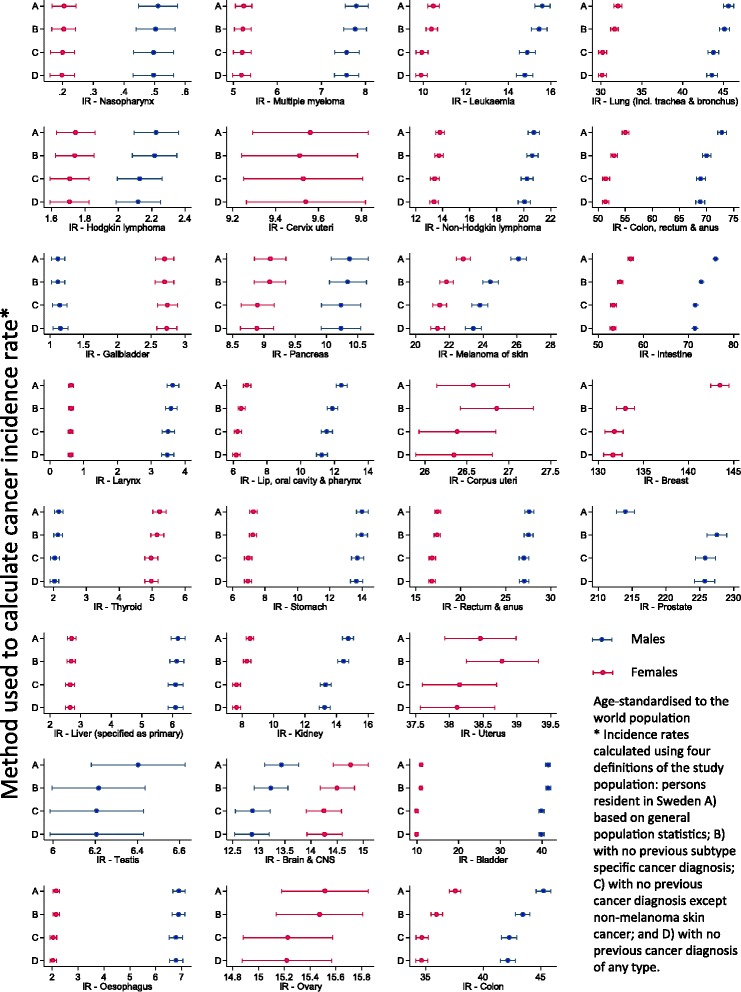
Age-standardized incidence rates (per 100,000 person-years) for 29 cancer subtypes. Incidence rates calculated using four different definitions of the study population

After age-standardization, aggregate population incidence rates were ≥5% higher than subtype-specific incidence rates for three cancer subtypes: lip, oral cavity, and pharynx; breast (in women); and melanoma of skin (Fig. 1 and Additional file 1: Table S1). The greatest difference was for breast cancer in women, for which age-standardized aggregate population incidence rates were 8% higher than subtype-specific incidence rates (IRD: 10.5/100,000 person-years). However, age-standardized aggregate population incidence rates were 6% lower than subtype-specific incidence rates for prostate cancer (IRD: -13.5/100,000 person-years) and 1% lower for corpus uteri and uterus cancer (IRD: -0.3/100,000 person-years) (Fig. 1 and Additional file 1: Table S1). We found no difference between age-standardized aggregate population incidence rates and subtype-specific incidence rates in men and women for cancers of nasopharynx, esophagus, stomach, liver, gallbladder, pancreas, and ovary, or for Hodgkin lymphoma, and multiple myeloma (Fig. 1 and Additional file 1: Table S1).

**Fig. 2:**
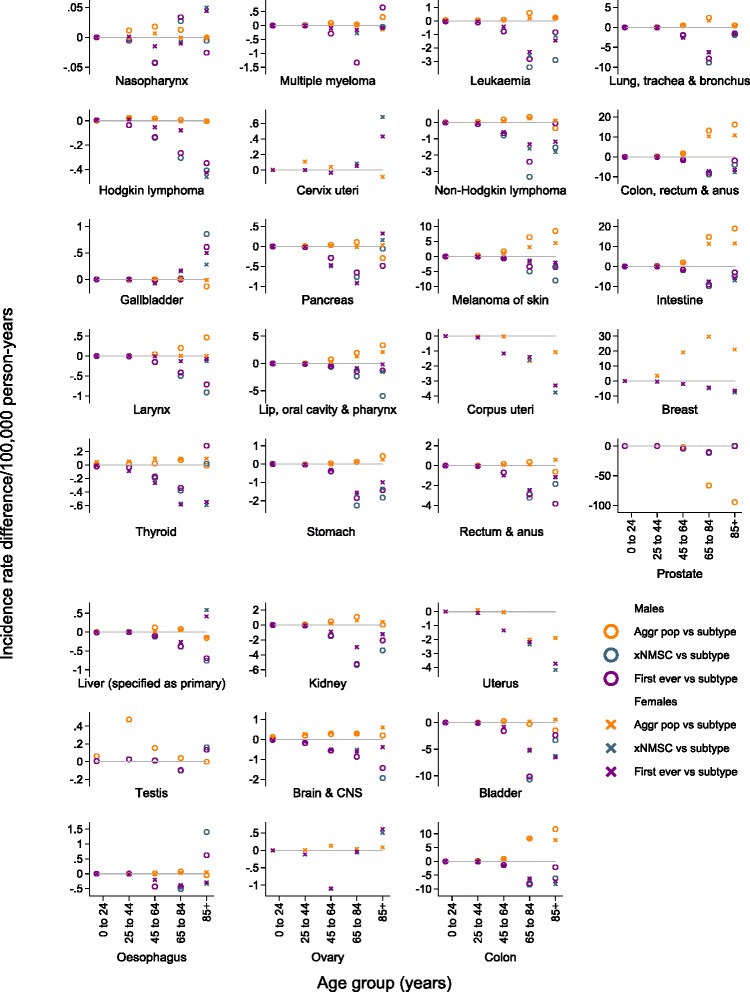
Age-group-specific incidence rate differences (per 100,000 person-years) for 29 different cancer subtypes. Incidence rates calculated comparing three different definitions of the study population (persons resident in Sweden, 1. based on general population statistics [Aggr pop]; 2. with no previous cancer diagnosis except non-melanoma skin cancer [xNMSC]; and 3. with no previous cancer diagnosis of any type [First ever]) to incidence rates based on a study population of persons resident in Sweden with no previous subtype-specific cancer diagnosis (Subtype)

**Fig. 3:**
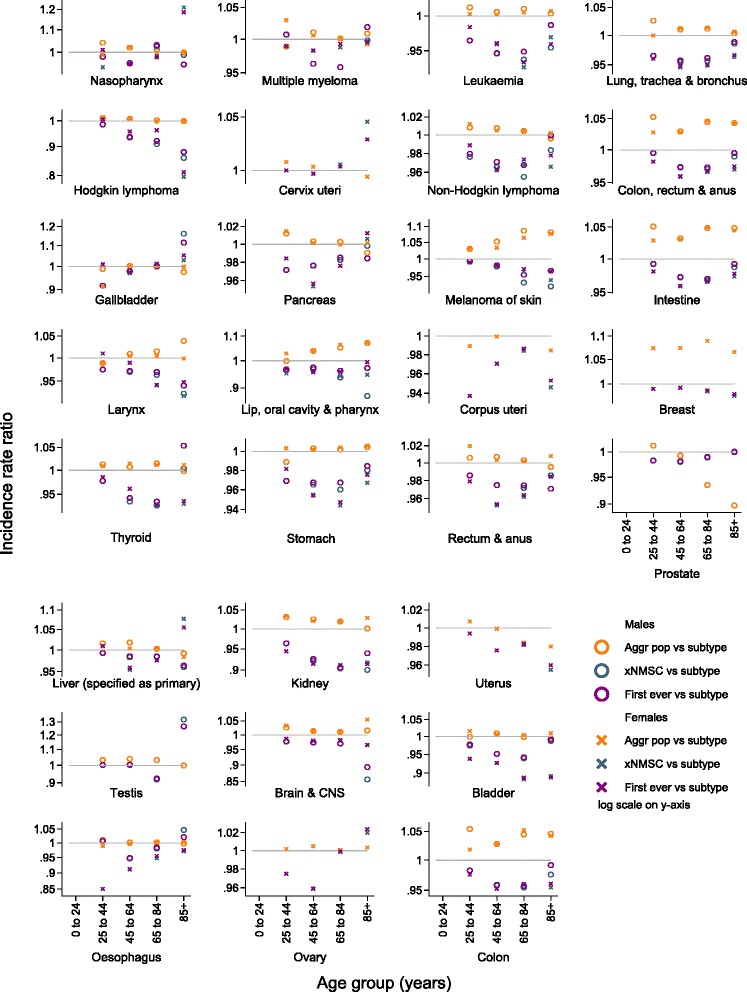
Age-group-specific incidence rate ratios for 29 different cancer subtypes. Incidence rates calculated comparing three different definitions of the study population (persons resident in Sweden, 1. based on general population statistics [Aggr pop]; 2. with no previous cancer diagnosis except non-melanoma skin cancer [xNMSC]; and 3. with no previous cancer diagnosis of any type [First ever]) to incidence rates based on a study population of persons resident in Sweden with no previous subtype-specific cancer diagnosis (Subtype) for 29 different cancer subtypes

Age-group-specific incidence rates primarily followed the patterns described above. The greatest absolute differences between methods reflected age-specific peaks in incidence rates (Fig. 2, Additional file 1: Tables S2 and S3). For example, the greatest difference between methods for testicular cancer was in the 25–44 years age group, while for lung, kidney, and breast cancers the greatest absolute difference was in the 65–84 years age group. For the most part the relative differences between aggregate population incidence rates and subtype-specific incidence rates were rather stable across age groups in both sexes (Fig. 3, Additional file 1: Tables S2 and S3).

### First cancer except non-melanoma skin cancer incidence rates compared to subtype-specific incidence rates

Incidence rates were similar for first cancer except non-melanoma skin cancer and first-ever cancer. As such, first cancer except non-melanoma skin cancer incidence rates compared to subtype-specific incidence rates reflect the results described below for first-ever cancer incidence rates compared to subtype-specific incidence rates.

### First-ever cancer incidence rates compared to subtype-specific incidence rates

Age-standardized first-ever cancer incidence rates were ≥5% lower than subtype-specific incidence rates for cancers of lip, oral cavity, and pharynx; esophagus (females); lung, trachea, and bronchus (females); kidney; bladder (females); and thyroid (males); and leukemia (females) (Fig. 1 and Additional file 1: Table S1). Despite this, the absolute difference in age-standardized incidence rates was less than 5 cases per 100,000 person-years for all subtypes (Fig. 1 and Additional file 1: Table S1).

Age-group-specific analyses indicated that, on an absolute scale, first-ever cancer incidence rates were often progressively lower than subtype-specific incidence rates in the 45–64 and 65–85 years age groups. For some cancer subtypes, for example, Hodgkin lymphoma, uterus, and breast cancers, this progression continued into the oldest age group. For other cancer subtypes, for example, lung and colorectal cancers, the absolute difference between these methods was reduced in the oldest age group, or the pattern was reversed (Fig. 2, Additional file 1: Tables S2 and S3). For most cancer subtypes the relative difference between first-ever cancer incidence rates and subtype-specific incidence rates across age groups followed a similar pattern to the absolute differences (Fig. 3, Additional file 1: Tables S2 and S3).

## Discussion

In a large population-based open cohort study, we highlight several important methodological factors that should be considered when calculating incidence rates. First, we demonstrate notable differences between incidence rates calculated from aggregate general population statistics compared to those based on individual-level data for some cancer subtypes. Second, we show that cancer incidence rates calculated from individual-level data vary depending on whether the study population includes individuals with a previous cancer diagnosis. However, for most cancer subtypes, these methods are broadly comparable. Although the results are only presented for Sweden in the period 2000–2010, these main findings are likely to be generalizable to other countries with similar social structure, distribution of cancer type in the general population, and cancer survival. Moreover, as social development results in older populations and better cancer survival, the importance of these methodological issues will become greater.

### Strengths and limitations

The main strengths of this study were the very large sample size and whole population coverage. The study also had some limitations. First, 40% of individuals included in the study were born before the cancer register in Sweden started (1958). However, 96.8% of these persons were younger than 40 years of age in 1958, so this will have little effect on the results for the period 2000–2010. In addition, we did not have information about cancer diagnoses before individuals immigrated to Sweden because cancers are registered in the country of diagnosis. However, the patterns of results were obtained when the analysis was restricted to individuals born in Sweden (Additional file 1: Table S4). In Sweden death-certificate-only and death-certificate-initiated cancer cases are not reported to the cancer register. However, since these data will be missing for all methods of calculating incidence rate, this underestimation will not impact the comparison between methods. There may be more advanced ways to correct the person-years at risk that were beyond the scope of the current paper but should be kept in mind. For example, excluding hysterectomized women from the risk population in calculations of uterus cancer or cholecystectomized persons from the risk population in calculations of gallbladder cancer. Finally, basal cell carcinoma has not been registered in Sweden so is one category of cancer that was not possible to include in this paper.

### Aggregate population incidence rates compared to subtype-specific incidence rates

Aggregate population incidence rates were higher than subtype-specific incidence rates for several cancer subtypes. For cancer subtypes that showed this pattern, excluding individuals with a previous subtype-specific cancer diagnosis from the study population reduced the numerator to a greater extent than the denominator. This can be explained if persons with a previous cancer diagnosis are more likely to have a subsequent diagnosis of the same cancer subtype than persons without a previous diagnosis of that subtype. Supporting this, the difference between these two methodologies was greatest for cancer subtypes with a higher chance of a second primary cancer of the same subtype, for example breast cancer in women [12] and colorectal cancer [13].

For prostate cancer aggregate population incidence rates were lower than subtype-specific incidence rates. As prostate cancer has a low fatality level there were many prevalent cases in the aggregate population statistics that were excluded when using individual-level data. As such, removing those with a previous subtype-specific cancer diagnosis reduced the denominator to a greater extent than the numerator.

For highly fatal cancers, such as pancreas cancer, we found no difference between aggregate population incidence rates and subtype-specific incidence rates, as expected. This is because there were very few prevalent cases to influence the denominator and a very low chance of a second diagnosis of the same subtype.

Differences between aggregate population incidence rates and subtype-specific incidence rates are important for two reasons. First, in planning health service provision, the use of aggregate population data is appropriate for most cancer subtypes, even if they are overestimated compared to individual-level data, as individuals with a second primary tumor of the same subtype still need access to health care despite their previous diagnosis. However, when aggregate population data underestimate incidence rates compared to individual-level data, there may be inadequate provision of services for individuals diagnosed with these cancer subtypes. Nonetheless, besides incidence rates health care planning is based on actual number of cases, so this issue may be minimized. Second, the effect of an exposure or intervention in a subpopulation with individual-level data can be examined using standardized incidence ratios. In such studies aggregate population statistics are often used to calculate the expected number of cases. Different methodologies for calculating incidence rates using the individual-level data compared to the aggregate population data will results in distortion of the standardized incidence ratios. In turn this may lead to important exposures being disregarded, while redundant interventions may be deemed effective, or vice versa.

### First-ever cancer, and first cancer except for non-melanoma skin cancer incidence rates compared to subtype-specific incidence rates

First-ever cancer, and first cancer except for non-melanoma skin cancer incidence rates were often lower than subtype-specific incidence rates. This can be explained since persons with a previous cancer diagnosis are more likely to have a subsequent cancer diagnosis than persons without a previous cancer diagnosis. For example, risk of subsequent neoplasm is raised in survivors of childhood cancer [14], and in adults diagnosed with first primary breast cancer (premenopausal), malignant melanomas, bladder, and head and neck cancers [15]. Increased risk of a second primary cancer may be related to ongoing surveillance of the patient leading to greater detection, subsequent cancers may be linked etiologically including via shared behavioral and genetic risk factors, and finally, treatment of the first malignancy may increase the risk of subsequent disease. However, the absolute difference between the methods for most cancer subtypes was small, particularly for age-standardized incidence rates. We therefore suggest that for most cancer subtypes the comparability between studies using different definitions of the study population is reasonable, especially if age-standardized rates are presented.

When studying cancer subtypes with greater differences between methods, careful consideration should be given to whether the previous cancer diagnosis is likely to be a confounder. If there is no reason to believe that the previous cancer is a confounder, then there is no reason to exclude individuals with a previous cancer. Our a priori hypothesis was that that there might be larger differences between incidence rates calculated with different study populations for leukemia, due to the increased risk of leukemia after treatment for a previous cancer subtype [16, 17]. However, there were not markedly greater differences between methodologies for leukemia than for other cancer subtypes. This indicates that persons with a previous cancer diagnosis may be more likely to have a subsequent cancer diagnosis than persons without a previous cancer diagnosis due to shared risk factors, rather than the previous cancer acting as a true confounder. As such, only excluding individuals with a previous cancer of the same subtype may often be the most appropriate way to define the study population. This is of particular relevance for studies with limited statistical power. Only excluding individuals with a previous subtype-specific cancer diagnosis, rather than all those with any previous cancer diagnosis, will increase the number of cases available for analysis and thus increase the statistical power.

### Age-group-specific incidence rates

Relative differences between aggregate population incidence rates and subtype-specific incidence rates were rather stable across age groups. In contrast, differences between first-ever cancer incidence rates and subtype-specific incidence rates varied by age group. The discussion above could therefore have a lesser or greater importance, depending on the age group being studied and the cancer outcome of interest.

## Conclusions

Cancer incidence rates vary depending on the definition of the study population. However, for most cancer subtypes, methods are broadly comparable when age-standardized incidence rates are considered. Nonetheless, when calculating cancer incidence rates one should consider the purpose of the information, the cancer outcome of interest, and the potential imprecision the choice of the numerator and the denominator might bring. This is particularly important if standardized incidence ratios are calculated based on general population statistics. The most appropriate definition of the study population depends on etiological arguments. However, defining the study population as individuals with no previous subtype-specific cancer diagnosis may be advantageous, particularly in studies with limited statistical power.

## Additional file

Additional file 1:
**Table S1.** Crude and age-standardized incidence rates (IR) (95% confidence intervals [95% CI]) per 100,000 person-years for 29 cancer subtypes in Swedish males and females between 2000 and 2010. IRs were calculated using four definitions of the study population: persons resident in Sweden 1) based on aggregate general population statistics (Aggr); 2) with no previous subtype-specific cancer diagnosis (subtype); 3) with no previous cancer diagnosis except non-melanoma skin cancer (xNMSC); and 4) with no previous cancer diagnosis of any type (First ever). IRs are presented with incidence rate differences (IRD) and incidence rate ratios (IRR) compared to subtype-specific IRs (ref). **Table S2.** Age-group-specific incidence rates (IR) (95% confidence intervals [95% CI]) per 100,000 person-years for 25 cancer subtypes in Swedish males between 2000 and 2010. IRs were calculated using four definitions of the study population: persons resident in Sweden 1) based on aggregate general population statistics (Aggr); 2) with no previous subtype-specific cancer diagnosis (subtype); 3) with no previous cancer diagnosis except non-melanoma skin cancer (xNMSC); and 4) with no previous cancer diagnosis of any type (First ever). IRs are presented with incidence rate differences (IRD) and incidence rate ratios (IRR) compared to subtype-specific IRs (ref). **Table S3.** Age-group-specific incidence rates (IR) (95% confidence intervals [95% CI]) per 100,000 person-years for 27 cancer subtypes in Swedish females between 2000 and 2010. IRs were calculated using four definitions of the study population: persons resident in Sweden 1) based on aggregate general population statistics (Aggr); 2) with no previous subtype-specific cancer diagnosis (subtype); 3) with no previous cancer diagnosis except non-melanoma skin cancer (xNMSC); and 4) with no previous cancer diagnosis of any type (First ever). IRs are presented with incidence rate differences (IRD) and incidence rate ratios (IRR) compared to subtype-specific IRs (ref). **Table S4.** Crude and age-standardized incidence rates (IR) (95% confidence intervals [95% CI]) per 100,000 person-years for 29 cancer subtypes in Swedish males and females between 2000 and 2010, including only individuals born in Sweden. IRs were calculated using four definitions of the study population: persons resident in Sweden 1) based on aggregate general population statistics (Aggr); 2) with no previous subtype-specific cancer diagnosis (subtype); 3) with no previous cancer diagnosis except non-melanoma skin cancer (xNMSC); and 4) with no previous cancer diagnosis of any type (First ever). IRs are presented with incidence rate differences (IRD) and incidence rate ratios (IRR) compared to subtype-specific IRs (ref). (DOCX 185 kb)

## Abbreviations

IRD: Incidence rate differences
IRR: Incidence rate ratios
