# Supplementary material for: Methodological choices affect cancer incidence rates: a cohort study
Source: Popul Health Metr. 2017 Jan 19;15:2. doi: 10.1186/s12963-017-0120-x (PMC5248500; doi:10.1186/s12963-017-0120-x)
Supplement: Additional file 1: — Table S1. Crude and age-standardized incidence rates (IR) (95% confidence intervals [95% CI]) per 100,000 person-years for 29 cancer subtypes in Swedish males and females between 2000 and 2010. IRs were calculated using four definitions of the study population: persons resident in Sweden 1) based on aggregate general population statistics (Aggr); 2) with no previous subtype-specific cancer diagnosis (subtype); 3) with no previous cancer diagnosis except non-melanoma skin cancer (xNMSC); and 4) with no previous cancer diagnosis of any type (First ever). IRs are presented with incidence rate differences (IRD) and incidence rate ratios (IRR) compared to subtype-specific IRs (ref). Table S2. Age-group-specific incidence rates (IR) (95% confidence intervals [95% CI]) per 100,000 person-years for 25 cancer subtypes in Swedish males between 2000 and 2010. IRs were calculated using four definitions of the study population: persons resident in Sweden 1) based on aggregate general population statistics (Aggr); 2) with no previous subtype-specific cancer diagnosis (subtype); 3) with no previous cancer diagnosis except non-melanoma skin cancer (xNMSC); and 4) with no previous cancer diagnosis of any type (First ever). IRs are presented with incidence rate differences (IRD) and incidence rate ratios (IRR) compared to subtype-specific IRs (ref). Table S3. Age-group-specific incidence rates (IR) (95% confidence intervals [95% CI]) per 100,000 person-years for 27 cancer subtypes in Swedish females between 2000 and 2010. IRs were calculated using four definitions of the study population: persons resident in Sweden 1) based on aggregate general population statistics (Aggr); 2) with no previous subtype-specific cancer diagnosis (subtype); 3) with no previous cancer diagnosis except non-melanoma skin cancer (xNMSC); and 4) with no previous cancer diagnosis of any type (First ever). IRs are presented with incidence rate differences (IRD) and incidence rate ratios (IRR) compared to subty [file 12963_2017_120_MOESM1_ESM.docx]

**Methodological choices affect cancer incidence rates: a cohort study**

*Population Health Metrics*

Hannah L. Brooke (corresponding author: hannah.brooke@ki.se)^1^, Mats Talbäck^1^, Maria Feychting^1^, Rickard Ljung^1^

^1^Unit of Epidemiology, Institute of Environmental Medicine, Karolinska Institutet, Stockholm, Sweden

**Supplementary Table 1:** Crude and age-standardized incidence rates (IR) (95% confidence intervals [95% CI]) per 100,000 person-years for 29 cancer subtypes in Swedish males and females between 2000 and 2010. IRs were calculated using four definitions of the study population: persons resident in Sweden 1) based on aggregate general population statistics (Aggr); 2) with no previous subtype-specific cancer diagnosis (subtype); 3) with no previous cancer diagnosis except non-melanoma skin cancer (xNMSC); and 4) with no previous cancer diagnosis of any type (First ever). IRs are presented with incidence rate differences (IRD) and incidence rate ratios (IRR) compared to subtype-specific IRs (ref).

| **Legend** | | |  | |  | | |  | |  | | |  |  | | | |  |  | | | |  |  | | |  | |  | | |
| --- | --- | --- | --- | --- | --- | --- | --- | --- | --- | --- | --- | --- | --- | --- | --- | --- | --- | --- | --- | --- | --- | --- | --- | --- | --- | --- | --- | --- | --- | --- | --- |
| Incidence rate difference (IRD) | | |  | | ≥10 higher than ref | | |  | | ≥5 higher than ref | | |  | <5 higher than ref | | | |  | <5 lower than ref | | | |  | ≥5 lower than ref | | |  | | ≥10 lower than ref | | |
| Incidence rate ratio (IRR) | | |  | | ≥10% higher than ref | | |  | | ≥5% higher than ref | | |  | <5% higher than ref | | | |  | <5% lower than ref | | | |  | ≥5% lower than ref | | |  | | ≥10% lower than ref | | |
|  |  | | | | |  |  | | | |  |  | | |  |  |  | | | |  |  | | | |  | |  | | |  |
|  | **Males** | | | | |  |  | | | |  |  | | |  |  | **Females** | | | |  |  | | | |  | |  | | |  |
|  | **Crude** | | | | |  | **Age-standardized Swedish population** | | | |  | **Age-standardized world population** | | |  |  | **Crude** | | | |  | **Age-standardized Swedish population** | | | |  | | **Age-standardized world population** | | |  |
|  | IR (95% CI) | | | IRD | | IRR | IR (95% CI) | | IRD | | IRR | IR (95% CI) | | | IRD | IRR | IR (95% CI) | | | IRD | IRR | IR (95% CI) | | | IRD | IRR | | IR (95% CI) | | IRD | IRR |
| C00-C14 **Lip, oral cavity, and pharynx** | | | | | | |  | |  | |  |  | | |  |  |  | | |  |  |  | | |  |  | |  | |  |  |
| Aggr | | 12.0 (11.7, 12.3) | | 0.5 | | 1.05 | 12.4 (12.1, 12.8) | | 0.5 | | 1.05 | 6.9 (6.7, 7.1) | | | 0.3 | 1.04 | 7.5 (7.3, 7.8) | | | 0.4 | 1.05 | 6.8 (6.6, 7.0) | | | 0.3 | 1.05 | | 3.8 (3.6, 3.9) | | 0.2 | 1.04 |
| Subtype | | 11.4 (11.2, 11.8) | | 0.0 | | 1.00 | 11.9 (11.6, 12.2) | | 0.0 | | 1.00 | 6.6 (6.4, 6.8) | | | 0.0 | 1.00 | 7.2 (6.9, 7.4) | | | 0.0 | 1.00 | 6.5 (6.3, 6.7) | | | 0.0 | 1.00 | | 3.6 (3.5, 3.8) | | 0.0 | 1.00 |
| xNMSC | | 10.5 (10.2, 10.8) | | -1.0 | | 0.91 | 11.6 (11.2, 11.9) | | -0.3 | | 0.97 | 6.4 (6.2, 6.6) | | | -0.2 | 0.97 | 6.5 (6.3, 6.7) | | | -0.7 | 0.91 | 6.3 (6.0, 6.5) | | | -0.2 | 0.97 | | 3.5 (3.4, 3.6) | | -0.1 | 0.97 |
| First ever | | 10.2 (9.9, 10.5) | | -1.2 | | 0.89 | 11.3 (10.9, 11.6) | | -0.6 | | 0.95 | 6.3 (6.1, 6.5) | | | -0.3 | 0.96 | 6.4 (6.1, 6.6) | | | -0.8 | 0.89 | 6.2 (6.0, 6.4) | | | -0.3 | 0.95 | | 3.5 (3.3, 3.6) | | -0.2 | 0.96 |
| C11 **Nasopharynx** | | | | | |  |  | |  | |  |  | | |  |  |  | | |  |  |  | | |  |  | |  | |  |  |
| Aggr | | 0.5 (0.5, 0.6) | | 0.0 | | 1.02 | 0.5 (0.4, 0.6) | | 0.0 | | 1.02 | 0.4 (0.3, 0.4) | | | 0.0 | 1.02 | 0.2 (0.2, 0.3) | | | 0.0 | 1.00 | 0.2 (0.2, 0.2) | | | 0.0 | 1.00 | | 0.2 (0.1, 0.2) | | 0.0 | 1.00 |
| Subtype | | 0.5 (0.4, 0.6) | | 0.0 | | 1.00 | 0.5 (0.4, 0.6) | | 0.0 | | 1.00 | 0.4 (0.3, 0.4) | | | 0.0 | 1.00 | 0.2 (0.2, 0.3) | | | 0.0 | 1.00 | 0.2 (0.2, 0.2) | | | 0.0 | 1.00 | | 0.1 (0.1, 0.2) | | 0.0 | 1.00 |
| xNMSC | | 0.5 (0.4, 0.5) | | 0.0 | | 0.95 | 0.5 (0.4, 0.6) | | 0.0 | | 0.99 | 0.4 (0.3, 0.4) | | | 0.0 | 0.98 | 0.2 (0.2, 0.2) | | | 0.0 | 0.95 | 0.2 (0.2, 0.2) | | | 0.0 | 0.98 | | 0.1 (0.1, 0.2) | | 0.0 | 0.99 |
| First ever | | 0.5 (0.4, 0.5) | | 0.0 | | 0.95 | 0.5 (0.4, 0.6) | | 0.0 | | 0.99 | 0.4 (0.3, 0.4) | | | 0.0 | 0.98 | 0.2 (0.2, 0.2) | | | 0.0 | 0.94 | 0.2 (0.2, 0.2) | | | 0.0 | 0.97 | | 0.1 (0.1, 0.2) | | 0.0 | 0.98 |
| C15 **Esophagus** | | | | | |  |  | |  | |  |  | | |  |  |  | | |  |  |  | | |  |  | |  | |  |  |
| Aggr | | 6.5 (6.3, 6.7) | | 0.0 | | 1.00 | 6.9 (6.7, 7.1) | | 0.0 | | 1.00 | 3.4 (3.3, 3.5) | | | 0.0 | 1.00 | 2.4 (2.3, 2.6) | | | 0.0 | 1.00 | 2.2 (2.0, 2.3) | | | 0.0 | 1.00 | | 1.0 (0.9, 1.1) | | 0.0 | 1.00 |
| Subtype | | 6.5 (6.3, 6.7) | | 0.0 | | 1.00 | 6.9 (6.6, 7.1) | | 0.0 | | 1.00 | 3.4 (3.3, 3.5) | | | 0.0 | 1.00 | 2.4 (2.3, 2.6) | | | 0.0 | 1.00 | 2.2 (2.0, 2.3) | | | 0.0 | 1.00 | | 1.0 (0.9, 1.1) | | 0.0 | 1.00 |
| xNMSC | | 5.9 (5.7, 6.1) | | -0.6 | | 0.90 | 6.8 (6.5, 7.0) | | -0.1 | | 0.99 | 3.3 (3.2, 3.4) | | | -0.1 | 0.98 | 2.1 (2.0, 2.3) | | | -0.3 | 0.87 | 2.1 (1.9, 2.2) | | | -0.1 | 0.95 | | 0.9 (0.9, 1.0) | | -0.1 | 0.93 |
| First ever | | 5.8 (5.6, 6.1) | | -0.7 | | 0.90 | 6.8 (6.5, 7.1) | | -0.1 | | 0.99 | 3.3 (3.2, 3.4) | | | -0.1 | 0.98 | 2.1 (2.0, 2.3) | | | -0.3 | 0.87 | 2.0 (1.9, 2.2) | | | -0.1 | 0.95 | | 0.9 (0.9, 1.0) | | -0.1 | 0.93 |
| C16 **Stomach** | | | | | |  |  | |  | |  |  | | |  |  |  | | |  |  |  | | |  |  | |  | |  |  |
| Aggr | | 12.7 (12.4, 13.0) | | 0.0 | | 1.00 | 14.0 (13.7, 14.4) | | 0.0 | | 1.00 | 6.1 (5.9, 6.3) | | | 0.0 | 1.00 | 8.2 (8.0, 8.5) | | | 0.0 | 1.00 | 7.3 (7.1, 7.5) | | | 0.0 | 1.00 | | 3.4 (3.2, 3.5) | | 0.0 | 1.00 |
| Subtype | | 12.7 (12.4, 13.0) | | 0.0 | | 1.00 | 14.0 (13.6, 14.3) | | 0.0 | | 1.00 | 6.1 (5.9, 6.2) | | | 0.0 | 1.00 | 8.2 (8.0, 8.5) | | | 0.0 | 1.00 | 7.2 (7.0, 7.5) | | | 0.0 | 1.00 | | 3.3 (3.2, 3.5) | | 0.0 | 1.00 |
| xNMSC | | 11.2 (10.9, 11.5) | | -1.4 | | 0.89 | 13.7 (13.3, 14.1) | | -0.3 | | 0.98 | 5.9 (5.8, 6.1) | | | -0.1 | 0.98 | 7.3 (7.0, 7.5) | | | -1.0 | 0.88 | 7.0 (6.7, 7.2) | | | -0.3 | 0.96 | | 3.2 (3.1, 3.3) | | -0.1 | 0.96 |
| First ever | | 11.1 (10.8, 11.4) | | -1.6 | | 0.88 | 13.7 (13.3, 14.0) | | -0.3 | | 0.98 | 5.9 (5.8, 6.1) | | | -0.2 | 0.97 | 7.2 (7.0, 7.5) | | | -1.0 | 0.88 | 6.9 (6.7, 7.2) | | | -0.3 | 0.96 | | 3.2 (3.1, 3.3) | | -0.1 | 0.96 |
| C17-C21 **Intestine** | | | | | |  |  | |  | |  |  | | |  |  |  | | |  |  |  | | |  |  | |  | |  |  |
| Aggr | | 69.5 (68.7, 70.2) | | 3.6 | | 1.05 | 76.1 (75.3, 76.9) | | 3.2 | | 1.04 | 34.2 (33.8, 34.5) | | | 1.3 | 1.04 | 64.4 (63.7, 65.1) | | | 3.2 | 1.05 | 57.2 (56.6, 57.8) | | | 2.4 | 1.04 | | 26.9 (26.6, 27.3) | | 1.0 | 1.04 |
| Subtype | | 65.8 (65.1, 66.6) | | 0.0 | | 1.00 | 72.9 (72.1, 73.7) | | 0.0 | | 1.00 | 32.8 (32.4, 33.2) | | | 0.0 | 1.00 | 61.1 (60.5, 61.8) | | | 0.0 | 1.00 | 54.8 (54.2, 55.5) | | | 0.0 | 1.00 | | 25.9 (25.6, 26.3) | | 0.0 | 1.00 |
| xNMSC | | 59.5 (58.8, 60.2) | | -6.3 | | 0.90 | 71.6 (70.7, 72.4) | | -1.3 | | 0.98 | 32.2 (31.8, 32.6) | | | -0.6 | 0.98 | 55.4 (54.7, 56.1) | | | -5.8 | 0.91 | 53.3 (52.7, 53.9) | | | -1.5 | 0.97 | | 25.2 (24.9, 25.5) | | -0.7 | 0.97 |
| First ever | | 59.0 (58.3, 59.6) | | -6.9 | | 0.90 | 71.5 (70.6, 72.3) | | -1.4 | | 0.98 | 32.2 (31.8, 32.6) | | | -0.6 | 0.98 | 55.0 (54.4, 55.7) | | | -6.1 | 0.90 | 53.2 (52.6, 53.9) | | | -1.6 | 0.97 | | 25.2 (24.8, 25.5) | | -0.7 | 0.97 |
| C18 **Colon** | | | |  | |  |  | |  | |  |  | | |  |  |  | | |  |  |  | | |  |  | |  | |  |  |
| Aggr | | 40.8 (40.3, 41.4) | | 1.8 | | 1.05 | 45.2 (44.6, 45.9) | | 1.8 | | 1.04 | 19.6 (19.3, 19.8) | | | 0.7 | 1.04 | 42.4 (41.9, 43.0) | | | 2.0 | 1.05 | 37.6 (37.1, 38.1) | | | 1.6 | 1.04 | | 17.1 (16.9, 17.4) | | 0.6 | 1.04 |
| Subtype | | 39.0 (38.4, 39.5) | | 0.0 | | 1.00 | 43.4 (42.8, 44.0) | | 0.0 | | 1.00 | 18.8 (18.5, 19.1) | | | 0.0 | 1.00 | 40.4 (39.9, 41.0) | | | 0.0 | 1.00 | 36.0 (35.5, 36.5) | | | 0.0 | 1.00 | | 16.5 (16.2, 16.8) | | 0.0 | 1.00 |
| xNMSC | | 34.5 (34.0, 35.0) | | -4.5 | | 0.89 | 42.2 (41.6, 42.9) | | -1.2 | | 0.97 | 18.3 (18.0, 18.6) | | | -0.5 | 0.97 | 36.1 (35.6, 36.6) | | | -4.3 | 0.89 | 34.7 (34.2, 35.2) | | | -1.3 | 0.96 | | 15.9 (15.7, 16.2) | | -0.5 | 0.97 |
| First ever | | 34.1 (33.6, 34.6) | | -4.9 | | 0.88 | 42.1 (41.5, 42.8) | | -1.3 | | 0.97 | 18.3 (18.0, 18.6) | | | -0.5 | 0.97 | 35.8 (35.3, 36.4) | | | -4.6 | 0.89 | 34.7 (34.1, 35.2) | | | -1.3 | 0.96 | | 15.9 (15.7, 16.2) | | -0.6 | 0.97 |
| C18-C21 **Colon, rectum, and anus** | | | | | | |  | |  | |  |  | | |  |  |  | | |  |  |  | | |  |  | |  | |  |  |
| Aggr | | 66.4 (65.7, 67.2) | | 3.2 | | 1.05 | 72.8 (72.1, 73.6) | | 2.8 | | 1.04 | 32.5 (32.2, 32.9) | | | 1.2 | 1.04 | 62.0 (61.3, 62.7) | | | 2.9 | 1.05 | 55.0 (54.4, 55.7) | | | 2.1 | 1.04 | | 25.8 (25.5, 26.2) | | 0.9 | 1.04 |
| Subtype | | 63.2 (62.5, 63.9) | | 0.0 | | 1.00 | 70.0 (69.2, 70.8) | | 0.0 | | 1.00 | 31.4 (31.0, 31.7) | | | 0.0 | 1.00 | 59.1 (58.4, 59.7) | | | 0.0 | 1.00 | 52.9 (52.3, 53.5) | | | 0.0 | 1.00 | | 24.9 (24.6, 25.3) | | 0.0 | 1.00 |
| xNMSC | | 57.2 (56.6, 57.9) | | -6.0 | | 0.91 | 69.0 (68.1, 69.8) | | -1.1 | | 0.98 | 30.9 (30.5, 31.3) | | | -0.5 | 0.99 | 53.5 (52.8, 54.1) | | | -5.6 | 0.91 | 51.4 (50.8, 52.1) | | | -1.5 | 0.97 | | 24.2 (23.9, 24.6) | | -0.7 | 0.97 |
| First ever | | 56.7 (56.0, 57.3) | | -6.5 | | 0.90 | 68.9 (68.0, 69.7) | | -1.2 | | 0.98 | 30.9 (30.5, 31.2) | | | -0.5 | 0.98 | 53.1 (52.4, 53.8) | | | -6.0 | 0.90 | 51.4 (50.7, 52.0) | | | -1.6 | 0.97 | | 24.2 (23.9, 24.6) | | -0.7 | 0.97 |
| C19-C21 **Rectum and anus** | | | |  | |  |  | |  | |  |  | | |  |  |  | | |  |  |  | | |  |  | |  | |  |  |
| Aggr | | 25.6 (25.2, 26.1) | | 0.2 | | 1.01 | 27.6 (27.1, 28.1) | | 0.1 | | 1.00 | 13.0 (12.7, 13.2) | | | 0.1 | 1.00 | 19.5 (19.1, 19.9) | | | 0.1 | 1.00 | 17.5 (17.1, 17.8) | | | 0.1 | 1.00 | | 8.7 (8.5, 8.9) | | 0.0 | 1.00 |
| Subtype | | 25.5 (25.0, 25.9) | | 0.0 | | 1.00 | 27.5 (27.0, 28.0) | | 0.0 | | 1.00 | 12.9 (12.7, 13.2) | | | 0.0 | 1.00 | 19.4 (19.1, 19.8) | | | 0.0 | 1.00 | 17.4 (17.1, 17.8) | | | 0.0 | 1.00 | | 8.7 (8.5, 8.9) | | 0.0 | 1.00 |
| xNMSC | | 23.0 (22.6, 23.4) | | -2.5 | | 0.90 | 27.0 (26.5, 27.5) | | -0.5 | | 0.98 | 12.7 (12.5, 13.0) | | | -0.2 | 0.98 | 17.5 (17.1, 17.9) | | | -1.9 | 0.90 | 16.9 (16.5, 17.2) | | | -0.5 | 0.97 | | 8.4 (8.2, 8.6) | | -0.3 | 0.97 |
| First ever | | 22.8 (22.4, 23.2) | | -2.7 | | 0.90 | 27.0 (26.5, 27.6) | | -0.5 | | 0.98 | 12.7 (12.5, 13.0) | | | -0.2 | 0.98 | 17.4 (17.0, 17.8) | | | -2.0 | 0.89 | 16.8 (16.5, 17.2) | | | -0.6 | 0.97 | | 8.4 (8.2, 8.6) | | -0.3 | 0.97 |
| C22 **Liver (specified as primary)** | | | | | |  |  | |  | |  |  | | |  |  |  | | |  |  |  | | |  |  | |  | |  |  |
| Aggr | | 5.8 (5.6, 6.0) | | 0.0 | | 1.01 | 6.2 (6.0, 6.4) | | 0.0 | | 1.01 | 3.1 (2.9, 3.2) | | | 0.0 | 1.01 | 3.0 (2.8, 3.1) | | | 0.0 | 1.00 | 2.7 (2.6, 2.8) | | | 0.0 | 1.00 | | 1.4 (1.3, 1.5) | | 0.0 | 1.01 |
| Subtype | | 5.7 (5.5, 6.0) | | 0.0 | | 1.00 | 6.1 (5.9, 6.4) | | 0.0 | | 1.00 | 3.0 (2.9, 3.2) | | | 0.0 | 1.00 | 3.0 (2.8, 3.1) | | | 0.0 | 1.00 | 2.7 (2.6, 2.8) | | | 0.0 | 1.00 | | 1.4 (1.3, 1.5) | | 0.0 | 1.00 |
| xNMSC | | 5.3 (5.0, 5.5) | | -0.5 | | 0.91 | 6.1 (5.9, 6.3) | | 0.0 | | 0.99 | 3.0 (2.9, 3.1) | | | 0.0 | 0.99 | 2.7 (2.6, 2.9) | | | -0.3 | 0.91 | 2.7 (2.5, 2.8) | | | 0.0 | 0.98 | | 1.4 (1.3, 1.5) | | 0.0 | 0.98 |
| First ever | | 5.2 (5.0, 5.4) | | -0.5 | | 0.91 | 6.1 (5.9, 6.3) | | 0.0 | | 0.99 | 3.0 (2.9, 3.1) | | | 0.0 | 0.99 | 2.7 (2.6, 2.9) | | | -0.3 | 0.91 | 2.7 (2.5, 2.8) | | | 0.0 | 0.98 | | 1.4 (1.3, 1.5) | | 0.0 | 0.98 |
| C23-C24 **Gallbladder** | | | | | |  |  | |  | |  |  | | |  |  |  | | |  |  |  | | |  |  | |  | |  |  |
| Aggr | | 1.0 (0.9, 1.1) | | 0.0 | | 1.00 | 1.1 (1.0, 1.2) | | 0.0 | | 1.00 | 0.5 (0.4, 0.5) | | | 0.0 | 1.00 | 3.0 (2.9, 3.2) | | | 0.0 | 1.00 | 2.7 (2.6, 2.8) | | | 0.0 | 1.00 | | 1.3 (1.2, 1.3) | | 0.0 | 1.00 |
| Subtype | | 1.0 (0.9, 1.1) | | 0.0 | | 1.00 | 1.1 (1.0, 1.2) | | 0.0 | | 1.00 | 0.5 (0.4, 0.5) | | | 0.0 | 1.00 | 3.0 (2.9, 3.2) | | | 0.0 | 1.00 | 2.7 (2.6, 2.8) | | | 0.0 | 1.00 | | 1.3 (1.2, 1.3) | | 0.0 | 1.00 |
| xNMSC | | 0.9 (0.9, 1.0) | | -0.1 | | 0.92 | 1.1 (1.0, 1.3) | | 0.0 | | 1.02 | 0.5 (0.4, 0.5) | | | 0.0 | 1.00 | 2.8 (2.7, 3.0) | | | -0.2 | 0.93 | 2.7 (2.6, 2.9) | | | 0.0 | 1.02 | | 1.3 (1.2, 1.3) | | 0.0 | 1.01 |
| First ever | | 0.9 (0.9, 1.0) | | -0.1 | | 0.91 | 1.2 (1.0, 1.3) | | 0.0 | | 1.03 | 0.5 (0.4, 0.5) | | | 0.0 | 1.00 | 2.8 (2.7, 3.0) | | | -0.2 | 0.92 | 2.7 (2.6, 2.9) | | | 0.0 | 1.01 | | 1.3 (1.2, 1.3) | | 0.0 | 1.01 |
| C25 **Pancreas** | | | | | |  |  | |  | |  |  | | |  |  |  | | |  |  |  | | |  |  | |  | |  |  |
| Aggr | | 9.8 (9.5, 10.1) | | 0.0 | | 1.00 | 10.4 (10.1, 10.7) | | 0.0 | | 1.00 | 5.2 (5.0, 5.3) | | | 0.0 | 1.00 | 10.1 (9.8, 10.3) | | | 0.0 | 1.00 | 9.1 (8.8, 9.4) | | | 0.0 | 1.00 | | 4.6 (4.4, 4.7) | | 0.0 | 1.00 |
| Subtype | | 9.8 (9.5, 10.1) | | 0.0 | | 1.00 | 10.3 (10.0, 10.6) | | 0.0 | | 1.00 | 5.1 (5.0, 5.3) | | | 0.0 | 1.00 | 10.1 (9.8, 10.4) | | | 0.0 | 1.00 | 9.1 (8.8, 9.3) | | | 0.0 | 1.00 | | 4.6 (4.4, 4.7) | | 0.0 | 1.00 |
| xNMSC | | 8.9 (8.7, 9.2) | | -0.9 | | 0.91 | 10.2 (9.9, 10.5) | | -0.1 | | 0.99 | 5.1 (4.9, 5.2) | | | -0.1 | 0.99 | 9.1 (8.9, 9.4) | | | -1.0 | 0.91 | 8.9 (8.6, 9.2) | | | -0.2 | 0.98 | | 4.5 (4.3, 4.6) | | -0.1 | 0.98 |
| First ever | | 8.9 (8.6, 9.1) | | -0.9 | | 0.90 | 10.2 (9.9, 10.6) | | -0.1 | | 0.99 | 5.1 (4.9, 5.2) | | | -0.1 | 0.99 | 9.1 (8.8, 9.4) | | | -1.0 | 0.90 | 8.9 (8.6, 9.2) | | | -0.2 | 0.98 | | 4.5 (4.3, 4.6) | | -0.1 | 0.98 |
| C32 **Larynx** | | | | | |  |  | |  | |  |  | | |  |  |  | | |  |  |  | | |  |  | |  | |  |  |
| Aggr | | 3.5 (3.3, 3.7) | | 0.0 | | 1.01 | 3.6 (3.5, 3.8) | | 0.1 | | 1.01 | 1.9 (1.8, 2.0) | | | 0.0 | 1.01 | 0.7 (0.6, 0.8) | | | 0.0 | 1.00 | 0.6 (0.6, 0.7) | | | 0.0 | 1.00 | | 0.4 (0.3, 0.4) | | 0.0 | 1.00 |
| Subtype | | 3.5 (3.3, 3.6) | | 0.0 | | 1.00 | 3.6 (3.4, 3.8) | | 0.0 | | 1.00 | 1.9 (1.8, 1.9) | | | 0.0 | 1.00 | 0.7 (0.6, 0.8) | | | 0.0 | 1.00 | 0.6 (0.6, 0.7) | | | 0.0 | 1.00 | | 0.4 (0.3, 0.4) | | 0.0 | 1.00 |
| xNMSC | | 3.1 (3.0, 3.3) | | -0.3 | | 0.91 | 3.5 (3.3, 3.7) | | -0.1 | | 0.98 | 1.8 (1.7, 1.9) | | | 0.0 | 0.98 | 0.6 (0.6, 0.7) | | | -0.1 | 0.91 | 0.6 (0.5, 0.7) | | | 0.0 | 0.97 | | 0.3 (0.3, 0.4) | | 0.0 | 0.98 |
| First ever | | 3.1 (2.9, 3.3) | | -0.4 | | 0.90 | 3.5 (3.3, 3.7) | | -0.1 | | 0.97 | 1.8 (1.7, 1.9) | | | 0.0 | 0.98 | 0.6 (0.6, 0.7) | | | -0.1 | 0.90 | 0.6 (0.5, 0.7) | | | 0.0 | 0.96 | | 0.3 (0.3, 0.4) | | 0.0 | 0.98 |
| C33-C34 **Lung (incl. trachea and bronchus)** | | | | | | | | |  | |  |  | | |  |  |  | | |  |  |  | | |  |  | |  | |  |  |
| Aggr | | 42.8 (42.2, 43.4) | | 0.5 | | 1.01 | 45.6 (45.0, 46.2) | | 0.5 | | 1.01 | 21.8 (21.5, 22.1) | | | 0.3 | 1.01 | 35.0 (34.5, 35.5) | | | 0.4 | 1.01 | 32.1 (31.6, 32.5) | | | 0.4 | 1.01 | | 17.6 (17.3, 17.9) | | 0.2 | 1.01 |
| Subtype | | 42.3 (41.8, 42.9) | | 0.0 | | 1.00 | 45.1 (44.5, 45.7) | | 0.0 | | 1.00 | 21.6 (21.2, 21.9) | | | 0.0 | 1.00 | 34.6 (34.1, 35.1) | | | 0.0 | 1.00 | 31.7 (31.2, 32.1) | | | 0.0 | 1.00 | | 17.3 (17.1, 17.6) | | 0.0 | 1.00 |
| xNMSC | | 37.6 (37.1, 38.2) | | -4.7 | | 0.89 | 43.8 (43.1, 44.4) | | -1.3 | | 0.97 | 20.9 (20.5, 21.2) | | | -0.7 | 0.97 | 30.9 (30.4, 31.4) | | | -3.7 | 0.89 | 30.2 (29.7, 30.7) | | | -1.5 | 0.95 | | 16.6 (16.3, 16.9) | | -0.8 | 0.96 |
| First ever | | 37.2 (36.7, 37.8) | | -5.1 | | 0.88 | 43.6 (42.9, 44.3) | | -1.5 | | 0.97 | 20.8 (20.5, 21.1) | | | -0.8 | 0.96 | 30.7 (30.2, 31.2) | | | -3.9 | 0.89 | 30.2 (29.7, 30.7) | | | -1.5 | 0.95 | | 16.6 (16.3, 16.8) | | -0.8 | 0.95 |
| C43 **Melanoma of skin** | | | | | | | | |  | |  |  | | |  |  |  | | |  |  |  | | |  |  | |  | |  |  |
| Aggr | | 24.9 (24.4, 25.3) | | 1.6 | | 1.07 | 26.1 (25.6, 26.6) | | 1.7 | | 1.07 | 14.6 (14.3, 14.9) | | | 0.8 | 1.06 | 24.3 (23.9, 24.7) | | | 1.1 | 1.05 | 22.8 (22.4, 23.2) | | | 1.0 | 1.05 | | 14.9 (14.6, 15.2) | | 0.5 | 1.04 |
| Subtype | | 23.3 (22.8, 23.7) | | 0.0 | | 1.00 | 24.4 (24.0, 24.9) | | 0.0 | | 1.00 | 13.8 (13.5, 14.0) | | | 0.0 | 1.00 | 23.2 (22.7, 23.6) | | | 0.0 | 1.00 | 21.8 (21.4, 22.2) | | | 0.0 | 1.00 | | 14.4 (14.1, 14.6) | | 0.0 | 1.00 |
| xNMSC | | 21.4 (21.0, 21.8) | | -1.8 | | 0.92 | 23.8 (23.3, 24.3) | | -0.6 | | 0.97 | 13.5 (13.2, 13.8) | | | -0.3 | 0.98 | 21.9 (21.4, 22.3) | | | -1.3 | 0.94 | 21.4 (21.0, 21.8) | | | -0.4 | 0.98 | | 14.1 (13.9, 14.4) | | -0.2 | 0.99 |
| First ever | | 21.0 (20.6, 21.5) | | -2.2 | | 0.90 | 23.4 (22.9, 23.9) | | -1.0 | | 0.96 | 13.4 (13.1, 13.6) | | | -0.4 | 0.97 | 21.7 (21.3, 22.1) | | | -1.5 | 0.94 | 21.3 (20.9, 21.7) | | | -0.5 | 0.98 | | 14.1 (13.8, 14.4) | | -0.3 | 0.98 |
| C50 **Breast** | | | | | | | | |  | |  |  | | |  |  |  | | |  |  |  | | |  |  | |  | |  |  |
| Aggr | | 0.9 (0.8, 1.0) | | 0.0 | | 1.00 | 0.9 (0.9, 1.0) | | 0.0 | | 1.01 | 0.4 (0.4, 0.5) | | | 0.0 | 1.01 | 154.2 (153.1, 155.2) | | | 13.8 | 1.10 | 143.5 (142.5, 144.5) | | | 10.5 | 1.08 | | 89.3 (88.6, 90.0) | | 6.4 | 1.08 |
| Subtype | | 0.9 (0.8, 0.9) | | 0.0 | | 1.00 | 0.9 (0.9, 1.0) | | 0.0 | | 1.00 | 0.4 (0.4, 0.5) | | | 0.0 | 1.00 | 140.4 (139.3, 141.4) | | | 0.0 | 1.00 | 133.0 (132.0, 134.0) | | | 0.0 | 1.00 | | 82.9 (82.3, 83.6) | | 0.0 | 1.00 |
| xNMSC | | 0.7 (0.7, 0.8) | | -0.1 | | 0.87 | 0.9 (0.8, 1.0) | | 0.0 | | 0.95 | 0.4 (0.4, 0.5) | | | 0.0 | 0.95 | 135.1 (134.0, 136.1) | | | -5.3 | 0.96 | 131.8 (130.7, 132.8) | | | -1.3 | 0.99 | | 82.3 (81.6, 83.0) | | -0.6 | 0.99 |
| First ever | | 0.7 (0.7, 0.8) | | -0.1 | | 0.85 | 0.9 (0.8, 1.0) | | -0.1 | | 0.94 | 0.4 (0.4, 0.5) | | | 0.0 | 0.94 | 134.6 (133.6, 135.7) | | | -5.8 | 0.96 | 131.6 (130.6, 132.6) | | | -1.4 | 0.99 | | 82.3 (81.6, 82.9) | | -0.7 | 0.99 |
| C53 **Cervix uteri** | | | | | | | | |  | |  |  | | |  |  |  | | |  |  |  | | |  |  | |  | |  |  |
| Aggr | |  | |  | |  |  | |  | |  |  | | |  |  | 9.8 (9.5, 10.1) | | | 0.1 | 1.01 | 9.6 (9.3, 9.8) | | | 0.0 | 1.01 | | 7.0 (6.7, 7.2) | | 0.0 | 1.01 |
| Subtype | |  | |  | |  |  | |  | |  |  | | |  |  | 9.7 (9.5, 10.0) | | | 0.0 | 1.00 | 9.5 (9.2, 9.8) | | | 0.0 | 1.00 | | 6.9 (6.7, 7.1) | | 0.0 | 1.00 |
| xNMSC | |  | |  | |  |  | |  | |  |  | | |  |  | 9.6 (9.3, 9.9) | | | -0.2 | 0.98 | 9.5 (9.3, 9.8) | | | 0.0 | 1.00 | | 6.9 (6.7, 7.1) | | 0.0 | 1.00 |
| First ever | |  | |  | |  |  | |  | |  |  | | |  |  | 9.6 (9.3, 9.9) | | | -0.2 | 0.98 | 9.5 (9.3, 9.8) | | | 0.0 | 1.00 | | 6.9 (6.7, 7.1) | | 0.0 | 1.00 |
| C53-C55 **Uterus** | | | | | | | | |  | |  |  | | |  |  |  | | |  |  |  | | |  |  | |  | |  |  |
| Aggr | |  | |  | |  |  | |  | |  |  | | |  |  | 41.6 (41.1, 42.2) | | | -0.1 | 1.00 | 38.5 (37.9, 39.0) | | | -0.3 | 0.99 | | 22.5 (22.1, 22.8) | | -0.1 | 1.00 |
| Subtype | |  | |  | |  |  | |  | |  |  | | |  |  | 41.7 (41.1, 42.3) | | | 0.0 | 1.00 | 38.8 (38.3, 39.3) | | | 0.0 | 1.00 | | 22.6 (22.2, 22.9) | | 0.0 | 1.00 |
| xNMSC | |  | |  | |  |  | |  | |  |  | | |  |  | 39.0 (38.4, 39.6) | | | -2.7 | 0.94 | 38.1 (37.6, 38.7) | | | -0.6 | 0.98 | | 22.3 (21.9, 22.6) | | -0.3 | 0.99 |
| First ever | |  | |  | |  |  | |  | |  |  | | |  |  | 38.9 (38.3, 39.4) | | | -2.8 | 0.93 | 38.1 (37.6, 38.7) | | | -0.7 | 0.98 | | 22.2 (21.9, 22.6) | | -0.3 | 0.99 |
| C54 **Corpus uteri** | | | | | |  |  | |  | |  |  | | |  |  |  | | |  |  |  | | |  |  | |  | |  |  |
| Aggr | |  | |  | |  |  | |  | |  |  | | |  |  | 29.3 (28.8, 29.8) | | | -0.2 | 0.99 | 26.6 (26.1, 27.0) | | | -0.3 | 0.99 | | 14.2 (13.9, 14.4) | | -0.1 | 0.99 |
| Subtype | |  | |  | |  |  | |  | |  |  | | |  |  | 29.5 (29.0, 29.9) | | | 0.0 | 1.00 | 26.9 (26.4, 27.3) | | | 0.0 | 1.00 | | 14.3 (14.0, 14.5) | | 0.0 | 1.00 |
| xNMSC | |  | |  | |  |  | |  | |  |  | | |  |  | 27.1 (26.7, 27.6) | | | -2.3 | 0.92 | 26.4 (25.9, 26.8) | | | -0.5 | 0.98 | | 14.0 (13.8, 14.3) | | -0.2 | 0.98 |
| First ever | |  | |  | |  |  | |  | |  |  | | |  |  | 27.0 (26.5, 27.5) | | | -2.5 | 0.92 | 26.3 (25.9, 26.8) | | | -0.5 | 0.98 | | 14.0 (13.8, 14.3) | | -0.2 | 0.98 |
| C56 **Ovary** | | | |  | |  |  | |  | |  |  | | |  |  |  | | |  |  |  | | |  |  | |  | |  |  |
| Aggr | |  | |  | |  |  | |  | |  |  | | |  |  | 16.6 (16.3, 17.0) | | | 0.1 | 1.00 | 15.5 (15.2, 15.8) | | | 0.0 | 1.00 | | 9.5 (9.3, 9.7) | | 0.0 | 1.00 |
| Subtype | |  | |  | |  |  | |  | |  |  | | |  |  | 16.6 (16.2, 16.9) | | | 0.0 | 1.00 | 15.5 (15.1, 15.8) | | | 0.0 | 1.00 | | 9.5 (9.2, 9.7) | | 0.0 | 1.00 |
| xNMSC | |  | |  | |  |  | |  | |  |  | | |  |  | 15.5 (15.2, 15.9) | | | -1.1 | 0.94 | 15.2 (14.9, 15.6) | | | -0.2 | 0.98 | | 9.3 (9.0, 9.5) | | -0.2 | 0.98 |
| First ever | |  | |  | |  |  | |  | |  |  | | |  |  | 15.5 (15.1, 15.8) | | | -1.1 | 0.93 | 15.2 (14.9, 15.6) | | | -0.3 | 0.98 | | 9.3 (9.0, 9.5) | | -0.2 | 0.98 |
| C61 **Prostate** | | | |  | |  |  | |  | |  |  | | |  |  |  | | |  |  |  | | |  |  | |  | |  |  |
| Aggr | | 201.9 (200.7, 203.2) | | -2.9 | | 0.99 | 214.0 (212.7, 215.4) | | -13.5 | | 0.94 | 102.1 (101.4, 102.8) | | | -4.8 | 0.96 |  | | |  |  |  | | |  |  | |  | |  |  |
| Subtype | | 204.8 (203.5, 206.1) | | 0.0 | | 1.00 | 227.5 (226.1, 229.0) | | 0.0 | | 1.00 | 106.9 (106.2, 107.6) | | | 0.0 | 1.00 |  | | |  |  |  | | |  |  | |  | |  |  |
| xNMSC | | 193.6 (192.4, 194.9) | | -11.2 | | 0.95 | 225.8 (224.3, 227.3) | | -1.7 | | 0.99 | 106.0 (105.3, 106.7) | | | -0.9 | 0.99 |  | | |  |  |  | | |  |  | |  | |  |  |
| First ever | | 192.1 (190.8, 193.3) | | -12.7 | | 0.94 | 225.7 (224.2, 227.2) | | -1.8 | | 0.99 | 106.0 (105.3, 106.7) | | | -1.0 | 0.99 |  | | |  |  |  | | |  |  | |  | |  |  |
| C62 **Testis** | | | |  | |  |  | |  | |  |  | | |  |  |  | | |  |  |  | | |  |  | |  | |  |  |
| Aggr | | 6.4 (6.2, 6.7) | | 0.2 | | 1.04 | 6.4 (6.2, 6.6) | | 0.2 | | 1.03 | 6.3 (6.1, 6.5) | | | 0.2 | 1.03 |  | | |  |  |  | | |  |  | |  | |  |  |
| Subtype | | 6.2 (6.0, 6.5) | | 0.0 | | 1.00 | 6.2 (6.0, 6.4) | | 0.0 | | 1.00 | 6.1 (5.9, 6.3) | | | 0.0 | 1.00 |  | | |  |  |  | | |  |  | |  | |  |  |
| xNMSC | | 6.4 (6.1, 6.6) | | 0.1 | | 1.02 | 6.2 (6.0, 6.4) | | 0.0 | | 1.00 | 6.1 (5.9, 6.3) | | | 0.0 | 1.00 |  | | |  |  |  | | |  |  | |  | |  |  |
| First ever | | 6.4 (6.1, 6.6) | | 0.1 | | 1.02 | 6.2 (6.0, 6.4) | | 0.0 | | 1.00 | 6.1 (5.9, 6.3) | | | 0.0 | 1.00 |  | | |  |  |  | | |  |  | |  | |  |  |
| C64 **Kidney** | | | |  | |  |  | |  | |  |  | | |  |  |  | | |  |  |  | | |  |  | |  | |  |  |
| Aggr | | 14.1 (13.8, 14.5) | | 0.3 | | 1.02 | 14.7 (14.4, 15.1) | | 0.3 | | 1.02 | 8.1 (7.9, 8.3) | | | 0.2 | 1.02 | 9.2 (9.0, 9.5) | | | 0.2 | 1.02 | 8.5 (8.2, 8.7) | | | 0.2 | 1.02 | | 4.8 (4.6, 4.9) | | 0.1 | 1.02 |
| Subtype | | 13.8 (13.5, 14.2) | | 0.0 | | 1.00 | 14.4 (14.1, 14.8) | | 0.0 | | 1.00 | 7.9 (7.7, 8.1) | | | 0.0 | 1.00 | 9.1 (8.8, 9.3) | | | 0.0 | 1.00 | 8.3 (8.1, 8.6) | | | 0.0 | 1.00 | | 4.7 (4.5, 4.8) | | 0.0 | 1.00 |
| xNMSC | | 11.9 (11.6, 12.2) | | -1.9 | | 0.86 | 13.3 (13.0, 13.7) | | -1.1 | | 0.92 | 7.3 (7.1, 7.5) | | | -0.6 | 0.93 | 7.8 (7.5, 8.0) | | | -1.3 | 0.86 | 7.7 (7.4, 7.9) | | | -0.7 | 0.92 | | 4.3 (4.2, 4.5) | | -0.3 | 0.93 |
| First ever | | 11.8 (11.5, 12.1) | | -2.0 | | 0.85 | 13.2 (12.9, 13.6) | | -1.2 | | 0.92 | 7.3 (7.1, 7.5) | | | -0.6 | 0.92 | 7.8 (7.5, 8.0) | | | -1.3 | 0.86 | 7.7 (7.4, 7.9) | | | -0.7 | 0.92 | | 4.3 (4.2, 4.5) | | -0.3 | 0.93 |
| C67 **Bladder** | | | |  | |  |  | |  | |  |  | | |  |  |  | | |  |  |  | | |  |  | |  | |  |  |
| Aggr | | 37.4 (36.9, 37.9) | | 0.3 | | 1.01 | 41.4 (40.8, 42.0) | | 0.0 | | 1.00 | 17.8 (17.5, 18.0) | | | 0.0 | 1.00 | 12.5 (12.2, 12.9) | | | 0.1 | 1.01 | 11.0 (10.8, 11.3) | | | 0.1 | 1.01 | | 5.1 (5.0, 5.3) | | 0.0 | 1.01 |
| Subtype | | 37.1 (36.6, 37.6) | | 0.0 | | 1.00 | 41.4 (40.8, 42.0) | | 0.0 | | 1.00 | 17.7 (17.5, 18.0) | | | 0.0 | 1.00 | 12.5 (12.2, 12.8) | | | 0.0 | 1.00 | 11.0 (10.7, 11.3) | | | 0.0 | 1.00 | | 5.1 (5.0, 5.3) | | 0.0 | 1.00 |
| xNMSC | | 32.5 (32.0, 33.0) | | -4.6 | | 0.88 | 39.8 (39.2, 40.5) | | -1.6 | | 0.96 | 17.0 (16.7, 17.3) | | | -0.7 | 0.96 | 10.4 (10.1, 10.7) | | | -2.1 | 0.83 | 9.9 (9.7, 10.2) | | | -1.0 | 0.90 | | 4.7 (4.5, 4.8) | | -0.4 | 0.92 |
| First ever | | 32.1 (31.6, 32.6) | | -5.0 | | 0.87 | 39.8 (39.1, 40.4) | | -1.7 | | 0.96 | 17.0 (16.7, 17.3) | | | -0.7 | 0.96 | 10.3 (10.0, 10.6) | | | -2.2 | 0.83 | 9.9 (9.6, 10.2) | | | -1.1 | 0.90 | | 4.7 (4.5, 4.8) | | -0.4 | 0.92 |
| C70-C72 **Brain and central nervous system** | | | | | | |  | |  | |  |  | | |  |  |  | | |  |  |  | | |  |  | |  | |  |  |
| Aggr | | 13.4 (13.1, 13.7) | | 0.2 | | 1.02 | 13.4 (13.1, 13.8) | | 0.2 | | 1.02 | 10.2 (9.9, 10.5) | | | 0.2 | 1.02 | 15.3 (15.0, 15.7) | | | 0.3 | 1.02 | 14.8 (14.4, 15.1) | | | 0.3 | 1.02 | | 11.1 (10.8, 11.4) | | 0.2 | 1.02 |
| Subtype | | 13.2 (12.9, 13.5) | | 0.0 | | 1.00 | 13.2 (12.9, 13.6) | | 0.0 | | 1.00 | 10.0 (9.7, 10.3) | | | 0.0 | 1.00 | 15.1 (14.7, 15.4) | | | 0.0 | 1.00 | 14.5 (14.2, 14.8) | | | 0.0 | 1.00 | | 10.9 (10.6, 11.2) | | 0.0 | 1.00 |
| xNMSC | | 12.5 (12.2, 12.9) | | -0.7 | | 0.95 | 12.9 (12.6, 13.2) | | -0.3 | | 0.97 | 9.8 (9.5, 10.1) | | | -0.2 | 0.98 | 14.4 (14.0, 14.7) | | | -0.7 | 0.95 | 14.2 (13.9, 14.6) | | | -0.3 | 0.98 | | 10.7 (10.5, 11.0) | | -0.2 | 0.98 |
| First ever | | 12.5 (12.2, 12.8) | | -0.7 | | 0.95 | 12.9 (12.5, 13.2) | | -0.4 | | 0.97 | 9.8 (9.5, 10.1) | | | -0.2 | 0.98 | 14.4 (14.0, 14.7) | | | -0.7 | 0.95 | 14.3 (13.9, 14.6) | | | -0.2 | 0.98 | | 10.7 (10.5, 11.0) | | -0.2 | 0.98 |
| C73 **Thyroid** | | | |  | |  |  | |  | |  |  | | |  |  |  | | |  |  |  | | |  |  | |  | |  |  |
| Aggr | | 2.1 (2.0, 2.2) | | 0.0 | | 1.01 | 2.2 (2.0, 2.3) | | 0.0 | | 1.01 | 1.4 (1.3, 1.5) | | | 0.0 | 1.01 | 5.4 (5.2, 5.6) | | | 0.1 | 1.01 | 5.2 (5.0, 5.4) | | | 0.1 | 1.01 | | 4.0 (3.8, 4.1) | | 0.1 | 1.01 |
| Subtype | | 2.1 (2.0, 2.2) | | 0.0 | | 1.00 | 2.1 (2.0, 2.3) | | 0.0 | | 1.00 | 1.4 (1.3, 1.5) | | | 0.0 | 1.00 | 5.3 (5.1, 5.5) | | | 0.0 | 1.00 | 5.2 (5.0, 5.4) | | | 0.0 | 1.00 | | 3.9 (3.7, 4.1) | | 0.0 | 1.00 |
| xNMSC | | 1.9 (1.8, 2.0) | | -0.2 | | 0.92 | 2.0 (1.9, 2.2) | | -0.1 | | 0.96 | 1.4 (1.3, 1.4) | | | -0.1 | 0.95 | 5.0 (4.8, 5.2) | | | -0.3 | 0.94 | 5.0 (4.8, 5.2) | | | -0.2 | 0.96 | | 3.8 (3.6, 4.0) | | -0.1 | 0.97 |
| First ever | | 1.9 (1.8, 2.0) | | -0.2 | | 0.91 | 2.0 (1.9, 2.2) | | -0.1 | | 0.95 | 1.3 (1.3, 1.4) | | | -0.1 | 0.95 | 5.0 (4.8, 5.2) | | | -0.3 | 0.94 | 5.0 (4.8, 5.2) | | | -0.2 | 0.96 | | 3.8 (3.6, 4.0) | | -0.1 | 0.97 |
| C81 **Hodgkin lymphoma** | | | | | |  |  | |  | |  |  | | |  |  |  | | |  |  |  | | |  |  | |  | |  |  |
| Aggr | | 2.2 (2.1, 2.3) | | 0.0 | | 1.01 | 2.2 (2.1, 2.4) | | 0.0 | | 1.00 | 1.9 (1.8, 2.0) | | | 0.0 | 1.00 | 1.8 (1.7, 1.9) | | | 0.0 | 1.01 | 1.7 (1.6, 1.9) | | | 0.0 | 1.00 | | 1.7 (1.5, 1.8) | | 0.0 | 1.00 |
| Subtype | | 2.2 (2.1, 2.3) | | 0.0 | | 1.00 | 2.2 (2.1, 2.3) | | 0.0 | | 1.00 | 1.9 (1.8, 2.0) | | | 0.0 | 1.00 | 1.8 (1.6, 1.9) | | | 0.0 | 1.00 | 1.7 (1.6, 1.9) | | | 0.0 | 1.00 | | 1.7 (1.5, 1.8) | | 0.0 | 1.00 |
| xNMSC | | 2.1 (2.0, 2.2) | | -0.1 | | 0.95 | 2.1 (2.0, 2.3) | | -0.1 | | 0.96 | 1.9 (1.7, 2.0) | | | 0.0 | 0.97 | 1.7 (1.6, 1.8) | | | 0.0 | 0.98 | 1.7 (1.6, 1.8) | | | 0.0 | 0.98 | | 1.6 (1.5, 1.8) | | 0.0 | 0.99 |
| First ever | | 2.1 (2.0, 2.2) | | -0.1 | | 0.95 | 2.1 (2.0, 2.3) | | -0.1 | | 0.96 | 1.9 (1.7, 2.0) | | | -0.1 | 0.97 | 1.7 (1.6, 1.8) | | | 0.0 | 0.98 | 1.7 (1.6, 1.8) | | | 0.0 | 0.98 | | 1.6 (1.5, 1.8) | | 0.0 | 0.99 |
| C82-C86 & C96 **Non-Hodgkin lymphoma** | | | | | | |  | |  | |  |  | | |  |  |  | | |  |  |  | | |  |  | |  | |  |  |
| Aggr | | 19.4 (19.0, 19.8) | | 0.1 | | 1.01 | 20.7 (20.3, 21.2) | | 0.1 | | 1.00 | 10.9 (10.6, 11.1) | | | 0.1 | 1.01 | 15.3 (14.9, 15.6) | | | 0.1 | 1.00 | 13.8 (13.5, 14.1) | | | 0.1 | 1.01 | | 7.4 (7.2, 7.6) | | 0.1 | 1.01 |
| Subtype | | 19.3 (18.9, 19.7) | | 0.0 | | 1.00 | 20.6 (20.2, 21.1) | | 0.0 | | 1.00 | 10.8 (10.6, 11.1) | | | 0.0 | 1.00 | 15.2 (14.8, 15.5) | | | 0.0 | 1.00 | 13.7 (13.4, 14.0) | | | 0.0 | 1.00 | | 7.4 (7.2, 7.6) | | 0.0 | 1.00 |
| xNMSC | | 17.6 (17.2, 17.9) | | -1.7 | | 0.91 | 20.2 (19.8, 20.7) | | -0.4 | | 0.98 | 10.6 (10.4, 10.8) | | | -0.2 | 0.98 | 13.8 (13.5, 14.2) | | | -1.3 | 0.91 | 13.4 (13.1, 13.8) | | | -0.3 | 0.98 | | 7.2 (7.0, 7.4) | | -0.1 | 0.98 |
| First ever | | 17.3 (16.9, 17.7) | | -2.0 | | 0.90 | 20.0 (19.6, 20.5) | | -0.6 | | 0.97 | 10.5 (10.3, 10.8) | | | -0.3 | 0.97 | 13.7 (13.4, 14.1) | | | -1.5 | 0.90 | 13.4 (13.0, 13.7) | | | -0.4 | 0.97 | | 7.2 (7.0, 7.4) | | -0.2 | 0.98 |
| C88 & C90 **Multiple myeloma** | | | | | | |  | |  | |  |  | | |  |  |  | | |  |  |  | | |  |  | |  | |  |  |
| Aggr | | 7.2 (7.0, 7.5) | | 0.0 | | 1.00 | 7.8 (7.5, 8.1) | | 0.0 | | 1.00 | 3.6 (3.5, 3.8) | | | 0.0 | 1.01 | 5.9 (5.6, 6.1) | | | 0.0 | 1.00 | 5.2 (5.0, 5.4) | | | 0.0 | 1.00 | | 2.5 (2.4, 2.6) | | 0.0 | 1.00 |
| Subtype | | 7.2 (7.0, 7.4) | | 0.0 | | 1.00 | 7.8 (7.5, 8.0) | | 0.0 | | 1.00 | 3.6 (3.5, 3.7) | | | 0.0 | 1.00 | 5.8 (5.6, 6.1) | | | 0.0 | 1.00 | 5.2 (5.0, 5.4) | | | 0.0 | 1.00 | | 2.5 (2.4, 2.6) | | 0.0 | 1.00 |
| xNMSC | | 6.4 (6.2, 6.6) | | -0.8 | | 0.89 | 7.6 (7.3, 7.9) | | -0.2 | | 0.98 | 3.5 (3.4, 3.6) | | | -0.1 | 0.97 | 5.4 (5.2, 5.6) | | | -0.5 | 0.92 | 5.2 (5.0, 5.4) | | | 0.0 | 1.00 | | 2.5 (2.4, 2.6) | | 0.0 | 1.00 |
| First ever | | 6.4 (6.1, 6.6) | | -0.8 | | 0.88 | 7.6 (7.3, 7.9) | | -0.2 | | 0.98 | 3.5 (3.4, 3.6) | | | -0.1 | 0.97 | 5.3 (5.1, 5.5) | | | -0.5 | 0.91 | 5.2 (5.0, 5.4) | | | 0.0 | 0.99 | | 2.5 (2.4, 2.6) | | 0.0 | 1.00 |
| C91-C95 **Leukemia** | | | | | | |  | |  | |  |  | | |  |  |  | | |  |  |  | | |  |  | |  | |  |  |
| Aggr | | 14.6 (14.2, 14.9) | | 0.1 | | 1.01 | 15.6 (15.2, 16.0) | | 0.1 | | 1.01 | 9.1 (8.9, 9.4) | | | 0.1 | 1.01 | 11.4 (11.1, 11.7) | | | 0.1 | 1.01 | 10.5 (10.2, 10.7) | | | 0.1 | 1.01 | | 6.8 (6.6, 7.0) | | 0.1 | 1.01 |
| Subtype | | 14.4 (14.1, 14.8) | | 0.0 | | 1.00 | 15.5 (15.1, 15.8) | | 0.0 | | 1.00 | 9.0 (8.8, 9.3) | | | 0.0 | 1.00 | 11.3 (11.1, 11.6) | | | 0.0 | 1.00 | 10.4 (10.1, 10.7) | | | 0.0 | 1.00 | | 6.7 (6.5, 7.0) | | 0.0 | 1.00 |
| xNMSC | | 12.9 (12.6, 13.2) | | -1.5 | | 0.90 | 14.9 (14.5, 15.3) | | -0.6 | | 0.96 | 8.7 (8.5, 9.0) | | | -0.3 | 0.97 | 10.2 (9.9, 10.5) | | | -1.1 | 0.90 | 9.9 (9.7, 10.2) | | | -0.5 | 0.96 | | 6.5 (6.3, 6.8) | | -0.2 | 0.97 |
| First ever | | 12.7 (12.4, 13.1) | | -1.7 | | 0.88 | 14.8 (14.4, 15.2) | | -0.7 | | 0.96 | 8.7 (8.4, 8.9) | | | -0.3 | 0.96 | 10.2 (9.9, 10.4) | | | -1.2 | 0.90 | 9.9 (9.6, 10.2) | | | -0.5 | 0.95 | | 6.5 (6.3, 6.7) | | -0.2 | 0.97 |

**Supplementary Table 2:** Age-group-specific incidence rates (IR) (95% confidence intervals [95% CI]) per 100,000 person-years for 25 cancer subtypes in Swedish males between 2000 and 2010. IRs were calculated using four definitions of the study population: persons resident in Sweden 1) based on aggregate general population statistics (Aggr); 2) with no previous subtype-specific cancer diagnosis (subtype); 3) with no previous cancer diagnosis except non-melanoma skin cancer (xNMSC); and 4) with no previous cancer diagnosis of any type (First ever). IRs are presented with incidence rate differences (IRD) and incidence rate ratios (IRR) compared to subtype-specific IRs (ref).

| **Legend** | | |  | |  | | | |  |  | | | |  |  | | |  | |  | |  |  | | | |  |  | | | |
| --- | --- | --- | --- | --- | --- | --- | --- | --- | --- | --- | --- | --- | --- | --- | --- | --- | --- | --- | --- | --- | --- | --- | --- | --- | --- | --- | --- | --- | --- | --- | --- |
| Incidence rate difference (IRD) | | |  | | ≥10 higher than ref | | | |  | ≥5 higher than ref | | | |  | <5 higher than ref | | |  | | <5 lower than ref | |  | ≥5 lower than ref | | | |  | ≥10 lower than ref | | | |
| Incidence rate ratio (IRR) | | |  | | ≥10% higher than ref | | | |  | ≥5% higher than ref | | | |  | <5% higher than ref | | |  | | <5% lower than ref | |  | ≥5% lower than ref | | | |  | ≥10% lower than ref | | | |
|  | |  | | | |  | |  | | | |  |  | | | |  | |  | | | | |  | |  | | | |  |  |
| **Males** | | **0–24 years** | | | |  | | **25–44 years** | | | |  | **45–64 years** | | | |  | | **65–84 years** | | | | |  | | **85+ years** | | | |  |  |
|  | | **IR (95% CI)** | | **IRD** | | **IRR** | | **IR (95% CI)** | | | **IRD** | **IRR** | **IR (95% CI)** | | | **IRD** | **IRR** | | **IR (95% CI)** | | **IRD** | | | **IRR** | | **IR (95% CI)** | | | **IRD** | **IRR** |  |
| C00-C14 **Lip, oral cavity, and pharynx** | | | | |  | |  | | |  |  |  | | |  |  | |  | |  | | | |  |  | | |  |  |  |  |
| Aggr | 0.2 (0.2, 0.3) | | 0.0 | | 1.06 | | 2.2 (2.0, 2.5) | | | 0.0 | 1.00 | 19.5 (18.7, 20.2) | | | 0.7 | 1.04 | | 39.3 (37.8, 40.8) | | 1.9 | | | | 1.05 | 49.9 (45.2, 55.0) | | | 3.3 | 1.07 |  |  |
| Subtype | 0.2 (0.2, 0.3) | | 0.0 | | 1.00 | | 2.2 (2.0, 2.5) | | | 0.0 | 1.00 | 18.7 (18.0, 19.5) | | | 0.0 | 1.00 | | 37.4 (35.9, 38.8) | | 0.0 | | | | 1.00 | 46.5 (42.0, 51.5) | | | 0.0 | 1.00 |  |  |
| xNMSC | 0.2 (0.2, 0.3) | | 0.0 | | 1.00 | | 2.1 (1.9, 2.4) | | | -0.1 | 0.97 | 18.2 (17.5, 19.0) | | | -0.5 | 0.97 | | 35.9 (34.4, 37.5) | | -1.4 | | | | 0.96 | 45.2 (40.2, 50.8) | | | -1.3 | 0.97 |  |  |
| First ever | 0.2 (0.2, 0.3) | | 0.0 | | 1.00 | | 2.1 (1.9, 2.4) | | | -0.1 | 0.96 | 18.1 (17.4, 18.9) | | | -0.6 | 0.97 | | 35.0 (33.5, 36.6) | | -2.3 | | | | 0.94 | 40.6 (35.8, 46.0) | | | -5.9 | 0.87 |  |  |
| C11 **Nasopharynx** | | | | |  | |  | | |  |  |  | | |  |  | |  | |  | | | |  |  | | |  |  |  |  |
| Aggr | 0.1 (0.0, 0.1) | | 0.0 | | 1.00 | | 0.3 (0.2, 0.4) | | | 0.0 | 1.04 | 0.9 (0.8, 1.1) | | | 0.0 | 1.02 | | 1.1 (0.9, 1.4) | | 0.0 | | | | 1.01 | 0.5 (0.2, 1.3) | | | 0.0 | 1.00 |  |  |
| Subtype | 0.1 (0.0, 0.1) | | 0.0 | | 1.00 | | 0.3 (0.2, 0.4) | | | 0.0 | 1.00 | 0.9 (0.8, 1.1) | | | 0.0 | 1.00 | | 1.1 (0.9, 1.4) | | 0.0 | | | | 1.00 | 0.5 (0.2, 1.4) | | | 0.0 | 1.00 |  |  |
| xNMSC | 0.1 (0.0, 0.1) | | 0.0 | | 1.00 | | 0.3 (0.2, 0.4) | | | 0.0 | 0.98 | 0.9 (0.7, 1.1) | | | 0.0 | 0.95 | | 1.1 (0.9, 1.4) | | 0.0 | | | | 1.03 | 0.5 (0.2, 1.5) | | | 0.0 | 0.95 |  |  |
| First ever | 0.1 (0.0, 0.1) | | 0.0 | | 1.00 | | 0.3 (0.2, 0.4) | | | 0.0 | 0.98 | 0.9 (0.7, 1.1) | | | 0.0 | 0.95 | | 1.1 (0.9, 1.4) | | 0.0 | | | | 1.02 | 0.5 (0.2, 1.6) | | | 0.0 | 0.99 |  |  |
| C15 **Esophagus** | | | | |  | |  | | |  |  |  | | |  |  | |  | |  | | | |  |  | | |  |  |  |  |
| Aggr | 0.0 (0.0, 0.0) | |  | |  | | 0.3 (0.3, 0.5) | | | 0.0 | 1.01 | 8.4 (7.9, 8.9) | | | 0.0 | 1.00 | | 27.2 (26.0, 28.5) | | 0.1 | | | | 1.00 | 30.1 (26.5, 34.2) | | | 0.0 | 1.00 |  |  |
| Subtype | 0.0 (0.0, 0.0) | |  | |  | | 0.3 (0.3, 0.5) | | | 0.0 | 1.00 | 8.4 (7.9, 8.9) | | | 0.0 | 1.00 | | 27.1 (25.9, 28.4) | | 0.0 | | | | 1.00 | 30.2 (26.6, 34.2) | | | 0.0 | 1.00 |  |  |
| xNMSC | 0.0 (0.0, 0.0) | |  | |  | | 0.3 (0.3, 0.5) | | | 0.0 | 1.01 | 7.9 (7.5, 8.5) | | | -0.4 | 0.95 | | 26.7 (25.4, 28.1) | | -0.4 | | | | 0.98 | 30.8 (26.7, 35.5) | | | 0.6 | 1.02 |  |  |
| First ever | 0.0 (0.0, 0.0) | |  | |  | | 0.3 (0.3, 0.5) | | | 0.0 | 1.01 | 7.9 (7.5, 8.4) | | | -0.4 | 0.95 | | 26.6 (25.3, 28.0) | | -0.5 | | | | 0.98 | 31.6 (27.4, 36.4) | | | 1.4 | 1.05 |  |  |
| C16 **Stomach** | | | | |  | |  | | |  |  |  | | |  |  | |  | |  | | | |  |  | | |  |  |  |  |
| Aggr | 0.0 (0.0, 0.1) | | 0.0 | | 1.00 | | 1.2 (1.0, 1.4) | | | 0.0 | 0.99 | 11.8 (11.3, 12.4) | | | 0.0 | 1.00 | | 56.8 (55.0, 58.6) | | 0.1 | | | | 1.00 | 92.1 (85.7, 99.1) | | | 0.4 | 1.00 |  |  |
| Subtype | 0.0 (0.0, 0.1) | | 0.0 | | 1.00 | | 1.2 (1.0, 1.4) | | | 0.0 | 1.00 | 11.8 (11.2, 12.4) | | | 0.0 | 1.00 | | 56.7 (54.9, 58.5) | | 0.0 | | | | 1.00 | 91.7 (85.2, 98.6) | | | 0.0 | 1.00 |  |  |
| xNMSC | 0.0 (0.0, 0.1) | | 0.0 | | 1.00 | | 1.1 (1.0, 1.3) | | | 0.0 | 0.97 | 11.4 (10.8, 12.0) | | | -0.4 | 0.97 | | 54.8 (53.0, 56.8) | | -1.8 | | | | 0.97 | 90.3 (83.1, 98.0) | | | -1.4 | 0.98 |  |  |
| First ever | 0.0 (0.0, 0.1) | | 0.0 | | 1.00 | | 1.1 (1.0, 1.3) | | | 0.0 | 0.97 | 11.4 (10.8, 12.0) | | | -0.4 | 0.97 | | 54.4 (52.6, 56.4) | | -2.3 | | | | 0.96 | 89.8 (82.6, 97.8) | | | -1.8 | 0.98 |  |  |
| C17-C21 **Intestine** | | |  | |  | |  | | |  |  |  | | |  |  | |  | |  | | | |  |  | | |  |  |  |  |
| Aggr | 0.5 (0.4, 0.6) | | 0.0 | | 1.02 | | 6.5 (6.1, 7.0) | | | 0.3 | 1.05 | 65.1 (63.7, 66.5) | | | 2.0 | 1.03 | | 320.5 (316.3, 324.8) | | 14.8 | | | | 1.05 | 412.0 (398.1, 426.4) | | | 19.1 | 1.05 |  |  |
| Subtype | 0.5 (0.4, 0.6) | | 0.0 | | 1.00 | | 6.2 (5.8, 6.6) | | | 0.0 | 1.00 | 63.1 (61.7, 64.4) | | | 0.0 | 1.00 | | 305.7 (301.5, 309.9) | | 0.0 | | | | 1.00 | 392.9 (379.0, 407.3) | | | 0.0 | 1.00 |  |  |
| xNMSC | 0.5 (0.4, 0.6) | | 0.0 | | 1.00 | | 6.2 (5.8, 6.6) | | | 0.0 | 0.99 | 61.3 (60.0, 62.7) | | | -1.7 | 0.97 | | 296.6 (292.2, 301.0) | | -9.1 | | | | 0.97 | 389.9 (374.7, 405.7) | | | -3.0 | 0.99 |  |  |
| First ever | 0.5 (0.4, 0.6) | | 0.0 | | 1.00 | | 6.2 (5.8, 6.6) | | | 0.0 | 0.99 | 61.3 (59.9, 62.7) | | | -1.8 | 0.97 | | 295.8 (291.4, 300.2) | | -9.9 | | | | 0.97 | 387.9 (372.5, 404.0) | | | -5.0 | 0.99 |  |  |
| C18 **Colon** |  | |  | |  | |  | | |  |  |  | | |  |  | |  | |  | | | |  |  | | |  |  |  |  |
| Aggr | 0.5 (0.4, 0.6) | | 0.0 | | 1.03 | | 3.9 (3.6, 4.2) | | | 0.2 | 1.05 | 33.7 (32.7, 34.7) | | | 0.9 | 1.03 | | 193.8 (190.5, 197.2) | | 8.3 | | | | 1.04 | 265.8 (254.7, 277.4) | | | 11.7 | 1.05 |  |  |
| Subtype | 0.5 (0.4, 0.6) | | 0.0 | | 1.00 | | 3.7 (3.4, 4.0) | | | 0.0 | 1.00 | 32.8 (31.8, 33.8) | | | 0.0 | 1.00 | | 185.5 (182.3, 188.8) | | 0.0 | | | | 1.00 | 254.2 (243.1, 265.7) | | | 0.0 | 1.00 |  |  |
| xNMSC | 0.5 (0.4, 0.6) | | 0.0 | | 1.00 | | 3.6 (3.3, 3.9) | | | -0.1 | 0.98 | 31.4 (30.4, 32.4) | | | -1.3 | 0.96 | | 177.5 (174.1, 181.0) | | -8.0 | | | | 0.96 | 252.0 (239.9, 264.8) | | | -2.1 | 0.99 |  |  |
| First ever | 0.5 (0.4, 0.6) | | 0.0 | | 1.00 | | 3.6 (3.3, 3.9) | | | -0.1 | 0.98 | 31.4 (30.4, 32.4) | | | -1.4 | 0.96 | | 177.0 (173.6, 180.5) | | -8.5 | | | | 0.95 | 248.0 (235.7, 260.9) | | | -6.2 | 0.98 |  |  |
| C18-C21 **Colon, rectum, and anus** | | | | |  | |  | | |  |  |  | | |  |  | |  | |  | | | |  |  | | |  |  |  |  |
| Aggr | 0.5 (0.4, 0.6) | | 0.0 | | 1.02 | | 6.0 (5.6, 6.4) | | | 0.3 | 1.05 | 61.3 (60.0, 62.7) | | | 1.7 | 1.03 | | 308.5 (304.3, 312.7) | | 13.1 | | | | 1.04 | 396.7 (383.0, 410.8) | | | 16.2 | 1.04 |  |  |
| Subtype | 0.5 (0.4, 0.6) | | 0.0 | | 1.00 | | 5.7 (5.3, 6.1) | | | 0.0 | 1.00 | 59.6 (58.2, 60.9) | | | 0.0 | 1.00 | | 295.3 (291.2, 299.5) | | 0.0 | | | | 1.00 | 380.4 (366.8, 394.6) | | | 0.0 | 1.00 |  |  |
| xNMSC | 0.5 (0.4, 0.6) | | 0.0 | | 1.00 | | 5.7 (5.3, 6.1) | | | 0.0 | 1.00 | 58.0 (56.7, 59.3) | | | -1.6 | 0.97 | | 287.4 (283.1, 291.8) | | -8.0 | | | | 0.97 | 378.5 (363.6, 394.1) | | | -1.9 | 0.99 |  |  |
| First ever | 0.5 (0.4, 0.6) | | 0.0 | | 1.00 | | 5.7 (5.3, 6.1) | | | 0.0 | 1.00 | 57.9 (56.6, 59.3) | | | -1.6 | 0.97 | | 286.6 (282.3, 291.0) | | -8.8 | | | | 0.97 | 376.4 (361.2, 392.3) | | | -4.0 | 0.99 |  |  |
| C19-C21 **Rectum and anus** | | | | |  | |  | | |  |  |  | | |  |  | |  | |  | | | |  |  | | |  |  |  |  |
| Aggr | 0.0 (0.0, 0.1) | | 0.0 | | 1.00 | | 2.1 (1.9, 2.4) | | | 0.0 | 1.01 | 27.6 (26.7, 28.5) | | | 0.2 | 1.01 | | 114.6 (112.1, 117.2) | | 0.4 | | | | 1.00 | 130.8 (123.1, 139.1) | | | -0.6 | 1.00 |  |  |
| Subtype | 0.0 (0.0, 0.1) | | 0.0 | | 1.00 | | 2.1 (1.9, 2.4) | | | 0.0 | 1.00 | 27.4 (26.5, 28.4) | | | 0.0 | 1.00 | | 114.3 (111.8, 116.9) | | 0.0 | | | | 1.00 | 131.4 (123.6, 139.7) | | | 0.0 | 1.00 |  |  |
| xNMSC | 0.0 (0.0, 0.1) | | 0.0 | | 1.00 | | 2.1 (1.9, 2.3) | | | 0.0 | 0.99 | 26.8 (25.9, 27.7) | | | -0.7 | 0.98 | | 111.4 (108.7, 114.1) | | -2.9 | | | | 0.97 | 127.6 (119.1, 136.8) | | | -3.8 | 0.97 |  |  |
| First ever | 0.0 (0.0, 0.1) | | 0.0 | | 1.00 | | 2.1 (1.9, 2.3) | | | 0.0 | 0.99 | 26.8 (25.9, 27.7) | | | -0.7 | 0.97 | | 111.1 (108.4, 113.8) | | -3.2 | | | | 0.97 | 129.6 (120.8, 139.0) | | | -1.8 | 0.99 |  |  |
| C22 **Liver (specified as primary)** | | | | |  | |  | | |  |  |  | | |  |  | |  | |  | | | |  |  | | |  |  |  |  |
| Aggr | 0.2 (0.1, 0.3) | | 0.0 | | 1.00 | | 0.5 (0.4, 0.7) | | | 0.0 | 1.02 | 6.9 (6.5, 7.4) | | | 0.1 | 1.02 | | 25.2 (24.0, 26.4) | | 0.1 | | | | 1.00 | 19.0 (16.2, 22.3) | | | -0.2 | 0.99 |  |  |
| Subtype | 0.2 (0.1, 0.3) | | 0.0 | | 1.00 | | 0.5 (0.4, 0.7) | | | 0.0 | 1.00 | 6.8 (6.4, 7.3) | | | 0.0 | 1.00 | | 25.1 (24.0, 26.4) | | 0.0 | | | | 1.00 | 19.1 (16.3, 22.4) | | | 0.0 | 1.00 |  |  |
| xNMSC | 0.2 (0.1, 0.3) | | 0.0 | | 0.96 | | 0.5 (0.4, 0.7) | | | 0.0 | 0.99 | 6.7 (6.3, 7.2) | | | -0.1 | 0.99 | | 24.8 (23.5, 26.1) | | -0.4 | | | | 0.98 | 18.4 (15.4, 22.1) | | | -0.7 | 0.96 |  |  |
| First ever | 0.2 (0.1, 0.3) | | 0.0 | | 0.96 | | 0.5 (0.4, 0.7) | | | 0.0 | 0.99 | 6.7 (6.3, 7.2) | | | -0.1 | 0.98 | | 24.8 (23.5, 26.1) | | -0.4 | | | | 0.99 | 18.4 (15.2, 22.1) | | | -0.8 | 0.96 |  |  |
| C2C-C24 **Gallbladder** | | |  | |  | |  | | |  |  |  | | |  |  | |  | |  | | | |  |  | | |  |  |  |  |
| Aggr | 0.0 (0.0, 0.0) | |  | |  | | 0.1 (0.0, 0.1) | | | 0.0 | 0.99 | 0.9 (0.8, 1.1) | | | 0.0 | 1.00 | | 5.0 (4.5, 5.5) | | 0.0 | | | | 1.00 | 5.2 (3.8, 7.0) | | | -0.1 | 0.97 |  |  |
| Subtype | 0.0 (0.0, 0.0) | |  | |  | | 0.1 (0.0, 0.1) | | | 0.0 | 1.00 | 0.9 (0.7, 1.1) | | | 0.0 | 1.00 | | 4.9 (4.4, 5.5) | | 0.0 | | | | 1.00 | 5.3 (3.9, 7.2) | | | 0.0 | 1.00 |  |  |
| xNMSC | 0.0 (0.0, 0.0) | |  | |  | | 0.1 (0.0, 0.1) | | | 0.0 | 0.92 | 0.9 (0.7, 1.1) | | | 0.0 | 0.98 | | 4.9 (4.4, 5.5) | | 0.0 | | | | 1.00 | 5.9 (4.3, 8.2) | | | 0.6 | 1.11 |  |  |
| First ever | 0.0 (0.0, 0.0) | |  | |  | | 0.1 (0.0, 0.1) | | | 0.0 | 0.92 | 0.9 (0.7, 1.1) | | | 0.0 | 0.98 | | 5.0 (4.4, 5.6) | | 0.0 | | | | 1.00 | 6.2 (4.5, 8.5) | | | 0.9 | 1.16 |  |  |
| C25 **Pancreas** | | |  | |  | |  | | |  |  |  | | |  |  | |  | |  | | | |  |  | | |  |  |  |  |
| Aggr | 0.0 (0.0, 0.1) | | 0.0 | | 1.20 | | 0.6 (0.5, 0.8) | | | 0.0 | 1.01 | 12.1 (11.5, 12.7) | | | 0.0 | 1.00 | | 43.7 (42.1, 45.3) | | 0.1 | | | | 1.00 | 30.5 (26.9, 34.6) | | | -0.3 | 0.99 |  |  |
| Subtype | 0.0 (0.0, 0.1) | | 0.0 | | 1.00 | | 0.6 (0.5, 0.8) | | | 0.0 | 1.00 | 12.0 (11.4, 12.6) | | | 0.0 | 1.00 | | 43.6 (42.0, 45.2) | | 0.0 | | | | 1.00 | 30.8 (27.1, 34.9) | | | 0.0 | 1.00 |  |  |
| xNMSC | 0.0 (0.0, 0.1) | | 0.0 | | 1.00 | | 0.6 (0.5, 0.8) | | | 0.0 | 0.97 | 11.7 (11.1, 12.3) | | | -0.3 | 0.98 | | 42.9 (41.3, 44.6) | | -0.7 | | | | 0.99 | 30.3 (26.3, 34.9) | | | -0.5 | 0.98 |  |  |
| First ever | 0.0 (0.0, 0.1) | | 0.0 | | 1.00 | | 0.6 (0.5, 0.8) | | | 0.0 | 0.97 | 11.7 (11.2, 12.4) | | | -0.3 | 0.98 | | 42.8 (41.2, 44.5) | | -0.8 | | | | 0.98 | 30.7 (26.6, 35.5) | | | -0.1 | 1.00 |  |  |
| C32 **Larynx** |  | |  | |  | |  | | |  |  |  | | |  |  | |  | |  | | | |  |  | | |  |  |  |  |
| Aggr | 0.0 (0.0, 0.0) | |  | |  | | 0.2 (0.2, 0.3) | | | 0.0 | 0.99 | 5.2 (4.8, 5.6) | | | 0.0 | 1.01 | | 13.7 (12.9, 14.7) | | 0.2 | | | | 1.02 | 12.4 (10.2, 15.1) | | | 0.5 | 1.04 |  |  |
| Subtype | 0.0 (0.0, 0.0) | |  | |  | | 0.2 (0.2, 0.3) | | | 0.0 | 1.00 | 5.1 (4.7, 5.5) | | | 0.0 | 1.00 | | 13.5 (12.7, 14.4) | | 0.0 | | | | 1.00 | 11.9 (9.7, 14.6) | | | 0.0 | 1.00 |  |  |
| xNMSC | 0.0 (0.0, 0.0) | |  | |  | | 0.2 (0.2, 0.3) | | | 0.0 | 0.97 | 5.0 (4.6, 5.4) | | | -0.1 | 0.97 | | 13.1 (12.2, 14.1) | | -0.4 | | | | 0.97 | 11.2 (8.9, 14.2) | | | -0.7 | 0.94 |  |  |
| First ever | 0.0 (0.0, 0.0) | |  | |  | | 0.2 (0.2, 0.3) | | | 0.0 | 0.97 | 5.0 (4.6, 5.4) | | | -0.2 | 0.97 | | 13.0 (12.1, 14.0) | | -0.5 | | | | 0.96 | 11.0 (8.7, 14.0) | | | -0.9 | 0.92 |  |  |
| C33-C34 **Lung (incl. trachea and bronchus)** | | | | | | |  | | |  |  |  | | |  |  | |  | |  | | | |  |  | | |  |  |  |  |
| Aggr | 0.1 (0.1, 0.2) | | 0.0 | | | 0.95 | 1.8 (1.6, 2.1) | | | 0.0 | 1.03 | 45.4 (44.3, 46.6) | | | 0.5 | 1.01 | | 206.1 (202.8, 209.6) | | 2.4 | | | | 1.01 | 133.4 (125.5, 141.7) | | | 0.5 | 1.00 |  |  |
| Subtype | 0.1 (0.1, 0.2) | | 0.0 | | | 1.00 | 1.8 (1.6, 2.0) | | | 0.0 | 1.00 | 45.0 (43.8, 46.1) | | | 0.0 | 1.00 | | 203.7 (200.4, 207.2) | | 0.0 | | | | 1.00 | 132.8 (125.0, 141.1) | | | 0.0 | 1.00 |  |  |
| xNMSC | 0.1 (0.1, 0.2) | | 0.0 | | | 0.95 | 1.7 (1.5, 1.9) | | | -0.1 | 0.96 | 43.0 (41.9, 44.2) | | | -1.9 | 0.96 | | 195.9 (192.3, 199.5) | | -7.9 | | | | 0.96 | 131.3 (122.6, 140.6) | | | -1.5 | 0.99 |  |  |
| First ever | 0.1 (0.1, 0.2) | | 0.0 | | | 0.95 | 1.7 (1.5, 1.9) | | | -0.1 | 0.97 | 42.9 (41.8, 44.1) | | | -2.0 | 0.95 | | 194.9 (191.3, 198.5) | | -8.8 | | | | 0.96 | 130.9 (122.1, 140.4) | | | -1.9 | 0.99 |  |  |
| C43 **Melanoma of skin** | | | | | | |  | | |  |  |  | | |  |  | |  | |  | | | |  |  | | |  |  |  |  |
| Aggr | 0.7 (0.6, 0.8) | | 0.0 | | | 1.04 | 11.0 (10.4, 11.5) | | | 0.3 | 1.03 | 33.6 (32.6, 34.6) | | | 1.7 | 1.05 | | 80.5 (78.4, 82.7) | | 6.4 | | | | 1.09 | 112.2 (105.1, 119.9) | | | 8.5 | 1.08 |  |  |
| Subtype | 0.7 (0.6, 0.8) | | 0.0 | | | 1.00 | 10.7 (10.1, 11.2) | | | 0.0 | 1.00 | 31.9 (30.9, 32.9) | | | 0.0 | 1.00 | | 74.1 (72.1, 76.2) | | 0.0 | | | | 1.00 | 103.7 (96.8, 111.1) | | | 0.0 | 1.00 |  |  |
| xNMSC | 0.7 (0.5, 0.8) | | 0.0 | | | 0.98 | 10.6 (10.0, 11.1) | | | -0.1 | 0.99 | 31.3 (30.3, 32.3) | | | -0.6 | 0.98 | | 70.7 (68.6, 72.9) | | -3.4 | | | | 0.95 | 100.2 (92.6, 108.4) | | | -3.5 | 0.97 |  |  |
| First ever | 0.7 (0.5, 0.8) | | 0.0 | | | 0.98 | 10.6 (10.0, 11.1) | | | -0.1 | 0.99 | 31.2 (30.2, 32.2) | | | -0.7 | 0.98 | | 69.1 (67.0, 71.3) | | -5.0 | | | | 0.93 | 95.7 (88.2, 103.9) | | | -8.1 | 0.92 |  |  |
| C50 **Breast** |  | |  | | |  |  | | |  |  |  | | |  |  | |  | |  | | | |  |  | | |  |  |  |  |
| Aggr | 0.0 (0.0, 0.1) | | 0.0 | | | 1.00 | 0.2 (0.1, 0.2) | | | 0.0 | 0.99 | 1.0 (0.9, 1.2) | | | 0.0 | 1.00 | | 3.3 (2.9, 3.8) | | 0.0 | | | | 1.01 | 6.1 (4.6, 8.1) | | | 0.1 | 1.02 |  |  |
| Subtype | 0.0 (0.0, 0.1) | | 0.0 | | | 1.00 | 0.2 (0.1, 0.2) | | | 0.0 | 1.00 | 1.0 (0.9, 1.2) | | | 0.0 | 1.00 | | 3.3 (2.9, 3.7) | | 0.0 | | | | 1.00 | 6.0 (4.5, 7.9) | | | 0.0 | 1.00 |  |  |
| xNMSC | 0.0 (0.0, 0.1) | | 0.0 | | | 1.00 | 0.1 (0.1, 0.2) | | | 0.0 | 0.96 | 1.0 (0.8, 1.2) | | | -0.1 | 0.94 | | 3.0 (2.5, 3.4) | | -0.3 | | | | 0.90 | 6.4 (4.7, 8.7) | | | 0.5 | 1.08 |  |  |
| First ever | 0.0 (0.0, 0.1) | | 0.0 | | | 1.00 | 0.1 (0.1, 0.2) | | | 0.0 | 0.96 | 1.0 (0.8, 1.2) | | | -0.1 | 0.94 | | 2.9 (2.5, 3.4) | | -0.4 | | | | 0.88 | 6.3 (4.6, 8.7) | | | 0.4 | 1.07 |  |  |
| C61 **Prostate** | | | | | |  |  | | |  |  |  | | |  |  | |  | |  | | | |  |  | | |  |  |  |  |
| Aggr | 0.0 (0.0, 0.0) | |  | | |  | 0.9 (0.8, 1.1) | | | 0.0 | 1.01 | 218.9 (216.4, 221.4) | | | -1.6 | 0.99 | | 957.5 (950.1, 964.9) | | -66.3 | | | | 0.94 | 824.9 (805.2, 845.2) | | | -94.5 | 0.90 |  |  |
| Subtype | 0.0 (0.0, 0.0) | |  | | |  | 0.9 (0.8, 1.1) | | | 0.0 | 1.00 | 220.5 (218.0, 223.1) | | | 0.0 | 1.00 | | 1023.8 (1015.9, 1031.7) | | 0.0 | | | | 1.00 | 919.4 (897.3, 942.1) | | | 0.0 | 1.00 |  |  |
| xNMSC | 0.0 (0.0, 0.0) | |  | | |  | 0.9 (0.8, 1.1) | | | 0.0 | 0.98 | 216.4 (213.8, 219.0) | | | -4.1 | 0.98 | | 1013.5 (1005.4, 1021.7) | | -10.3 | | | | 0.99 | 919.1 (895.6, 943.2) | | | -0.3 | 1.00 |  |  |
| First ever | 0.0 (0.0, 0.0) | |  | | |  | 0.9 (0.8, 1.1) | | | 0.0 | 0.98 | 216.2 (213.6, 218.7) | | | -4.4 | 0.98 | | 1012.2 (1004.0, 1020.4) | | -11.6 | | | | 0.99 | 920.0 (896.0, 944.6) | | | 0.6 | 1.00 |  |  |
| C62 **Testis** |  | |  | | |  |  | | |  |  |  | | |  |  | |  | |  | | | |  |  | | |  |  |  |  |
| Aggr | 3.3 (3.0, 3.6) | | 0.1 | | | 1.02 | 15.1 (14.5, 15.8) | | | 0.5 | 1.03 | 4.1 (3.7, 4.4) | | | 0.2 | 1.04 | | 1.3 (1.1, 1.6) | | 0.0 | | | | 1.03 | 0.5 (0.2, 1.3) | | | 0.0 | 1.00 |  |  |
| Subtype | 3.2 (2.9, 3.5) | | 0.0 | | | 1.00 | 14.7 (14.0, 15.3) | | | 0.0 | 1.00 | 3.9 (3.6, 4.3) | | | 0.0 | 1.00 | | 1.3 (1.0, 1.6) | | 0.0 | | | | 1.00 | 0.5 (0.2, 1.4) | | | 0.0 | 1.00 |  |  |
| xNMSC | 3.2 (2.9, 3.5) | | 0.0 | | | 1.00 | 14.7 (14.1, 15.4) | | | 0.0 | 1.00 | 3.9 (3.6, 4.3) | | | 0.0 | 1.00 | | 1.2 (0.9, 1.5) | | -0.1 | | | | 0.92 | 0.6 (0.2, 1.7) | | | 0.1 | 1.27 |  |  |
| First ever | 3.2 (2.9, 3.5) | | 0.0 | | | 1.00 | 14.7 (14.1, 15.4) | | | 0.0 | 1.00 | 3.9 (3.6, 4.3) | | | 0.0 | 1.00 | | 1.2 (0.9, 1.5) | | -0.1 | | | | 0.92 | 0.7 (0.3, 1.8) | | | 0.2 | 1.32 |  |  |
| C64 **Kidney** |  | |  | | |  |  | | |  |  |  | | |  |  | |  | |  | | | |  |  | | |  |  |  |  |
| Aggr | 0.6 (0.5, 0.8) | | 0.0 | | | 1.02 | 2.0 (1.7, 2.2) | | | 0.1 | 1.03 | 19.5 (18.8, 20.3) | | | 0.5 | 1.03 | | 56.5 (54.7, 58.3) | | 1.1 | | | | 1.02 | 34.2 (30.3, 38.5) | | | 0.0 | 1.00 |  |  |
| Subtype | 0.6 (0.5, 0.8) | | 0.0 | | | 1.00 | 1.9 (1.7, 2.1) | | | 0.0 | 1.00 | 19.0 (18.3, 19.8) | | | 0.0 | 1.00 | | 55.4 (53.7, 57.2) | | 0.0 | | | | 1.00 | 34.1 (30.3, 38.5) | | | 0.0 | 1.00 |  |  |
| xNMSC | 0.6 (0.5, 0.7) | | 0.0 | | | 0.99 | 1.8 (1.6, 2.1) | | | -0.1 | 0.96 | 17.6 (16.9, 18.4) | | | -1.4 | 0.93 | | 50.2 (48.4, 52.0) | | -5.2 | | | | 0.91 | 32.1 (27.9, 36.8) | | | -2.1 | 0.94 |  |  |
| First ever | 0.6 (0.5, 0.7) | | 0.0 | | | 0.99 | 1.8 (1.6, 2.1) | | | -0.1 | 0.96 | 17.5 (16.8, 18.3) | | | -1.5 | 0.92 | | 50.1 (48.3, 52.0) | | -5.3 | | | | 0.90 | 30.7 (26.6, 35.5) | | | -3.4 | 0.90 |  |  |
| C67 **Bladder** | | |  | | |  |  | | |  |  |  | | |  |  | |  | |  | | | |  |  | | |  |  |  |  |
| Aggr | 0.1 (0.1, 0.2) | | 0.0 | | | 1.00 | 2.0 (1.8, 2.3) | | | 0.0 | 1.00 | 32.6 (31.6, 33.6) | | | 0.3 | 1.01 | | 175.8 (172.6, 178.9) | | -0.2 | | | | 1.00 | 262.5 (251.5, 274.1) | | | -1.5 | 0.99 |  |  |
| Subtype | 0.1 (0.1, 0.2) | | 0.0 | | | 1.00 | 2.0 (1.8, 2.3) | | | 0.0 | 1.00 | 32.3 (31.4, 33.3) | | | 0.0 | 1.00 | | 176.0 (172.8, 179.2) | | 0.0 | | | | 1.00 | 264.0 (252.7, 275.8) | | | 0.0 | 1.00 |  |  |
| xNMSC | 0.1 (0.1, 0.2) | | 0.0 | | | 1.00 | 2.0 (1.8, 2.2) | | | 0.0 | 0.98 | 30.8 (29.8, 31.7) | | | -1.6 | 0.95 | | 165.9 (162.6, 169.2) | | -10.1 | | | | 0.94 | 261.6 (249.3, 274.6) | | | -2.4 | 0.99 |  |  |
| First ever | 0.1 (0.1, 0.2) | | 0.0 | | | 1.00 | 2.0 (1.8, 2.2) | | | -0.1 | 0.97 | 30.8 (29.8, 31.7) | | | -1.6 | 0.95 | | 165.3 (162.0, 168.7) | | -10.7 | | | | 0.94 | 260.7 (248.1, 273.9) | | | -3.3 | 0.99 |  |  |
| C70-C72 **Brain and central nervous system** | | | | | | |  | | |  |  |  | | |  |  | |  | |  | | | |  |  | | |  |  |  |  |
| Aggr | 4.5 (4.2, 4.8) | | 0.1 | | | 1.03 | 8.0 (7.5, 8.5) | | | 0.2 | 1.03 | 21.2 (20.4, 22.0) | | | 0.3 | 1.01 | | 29.6 (28.3, 30.9) | | 0.3 | | | | 1.01 | 13.7 (11.3, 16.5) | | | 0.2 | 1.02 |  |  |
| Subtype | 4.4 (4.0, 4.7) | | 0.0 | | | 1.00 | 7.8 (7.3, 8.3) | | | 0.0 | 1.00 | 20.9 (20.1, 21.7) | | | 0.0 | 1.00 | | 29.3 (28.0, 30.6) | | 0.0 | | | | 1.00 | 13.5 (11.1, 16.3) | | | 0.0 | 1.00 |  |  |
| xNMSC | 4.3 (4.0, 4.7) | | 0.0 | | | 1.00 | 7.6 (7.2, 8.1) | | | -0.2 | 0.98 | 20.4 (19.6, 21.2) | | | -0.5 | 0.97 | | 28.4 (27.1, 29.8) | | -0.9 | | | | 0.97 | 12.0 (9.6, 15.1) | | | -1.4 | 0.89 |  |  |
| First ever | 4.3 (4.0, 4.7) | | 0.0 | | | 1.00 | 7.6 (7.2, 8.1) | | | -0.2 | 0.98 | 20.4 (19.6, 21.2) | | | -0.6 | 0.97 | | 28.4 (27.1, 29.8) | | -0.9 | | | | 0.97 | 11.5 (9.1, 14.6) | | | -1.9 | 0.86 |  |  |
| C73 **Thyroid** |  | |  | | |  |  | | |  |  |  | | |  |  | |  | |  | | | |  |  | | |  |  |  |  |
| Aggr | 0.3 (0.2, 0.4) | | 0.0 | | | 1.02 | 1.5 (1.3, 1.8) | | | 0.0 | 1.01 | 3.0 (2.7, 3.3) | | | 0.0 | 1.01 | | 5.3 (4.8, 5.9) | | 0.1 | | | | 1.01 | 5.3 (3.9, 7.2) | | | 0.0 | 1.00 |  |  |
| Subtype | 0.3 (0.2, 0.4) | | 0.0 | | | 1.00 | 1.5 (1.3, 1.7) | | | 0.0 | 1.00 | 3.0 (2.7, 3.3) | | | 0.0 | 1.00 | | 5.2 (4.7, 5.8) | | 0.0 | | | | 1.00 | 5.3 (3.9, 7.2) | | | 0.0 | 1.00 |  |  |
| xNMSC | 0.3 (0.2, 0.4) | | 0.0 | | | 0.93 | 1.5 (1.3, 1.7) | | | 0.0 | 0.98 | 2.8 (2.5, 3.1) | | | -0.2 | 0.94 | | 4.9 (4.3, 5.5) | | -0.3 | | | | 0.94 | 5.6 (4.0, 7.8) | | | 0.3 | 1.05 |  |  |
| First ever | 0.3 (0.2, 0.4) | | 0.0 | | | 0.93 | 1.5 (1.3, 1.7) | | | 0.0 | 0.98 | 2.8 (2.5, 3.1) | | | -0.2 | 0.94 | | 4.8 (4.3, 5.4) | | -0.4 | | | | 0.93 | 5.3 (3.8, 7.6) | | | 0.0 | 1.00 |  |  |
| C81 **Hodgkin lymphoma** | | | | | |  |  | | |  |  |  | | |  |  | |  | |  | | | |  |  | | |  |  |  |  |
| Aggr | 1.5 (1.3, 1.7) | | 0.0 | | | 1.00 | 2.5 (2.2, 2.7) | | | 0.0 | 1.01 | 2.1 (1.9, 2.4) | | | 0.0 | 1.01 | | 3.4 (3.0, 3.8) | | 0.0 | | | | 1.00 | 2.9 (1.9, 4.4) | | | 0.0 | 1.00 |  |  |
| Subtype | 1.5 (1.3, 1.7) | | 0.0 | | | 1.00 | 2.4 (2.2, 2.7) | | | 0.0 | 1.00 | 2.1 (1.9, 2.4) | | | 0.0 | 1.00 | | 3.4 (3.0, 3.8) | | 0.0 | | | | 1.00 | 2.9 (1.9, 4.4) | | | 0.0 | 1.00 |  |  |
| xNMSC | 1.5 (1.3, 1.7) | | 0.0 | | | 1.00 | 2.4 (2.2, 2.7) | | | 0.0 | 0.99 | 2.0 (1.7, 2.2) | | | -0.1 | 0.94 | | 3.1 (2.7, 3.6) | | -0.3 | | | | 0.92 | 2.6 (1.6, 4.2) | | | -0.3 | 0.88 |  |  |
| First ever | 1.5 (1.3, 1.7) | | 0.0 | | | 1.00 | 2.4 (2.2, 2.7) | | | 0.0 | 0.99 | 2.0 (1.7, 2.2) | | | -0.1 | 0.93 | | 3.1 (2.6, 3.5) | | -0.3 | | | | 0.91 | 2.5 (1.5, 4.2) | | | -0.4 | 0.86 |  |  |
| C82-C86 & C96 **Non-Hodgkin lymphoma** | | | | | | |  | | |  |  |  | | |  |  | |  | |  | | | |  |  | | |  |  |  |  |
| Aggr | 1.6 (1.4, 1.8) | | 0.0 | | | 1.00 | 4.4 (4.1, 4.8) | | | 0.0 | 1.01 | 22.9 (22.1, 23.8) | | | 0.2 | 1.01 | | 74.6 (72.5, 76.7) | | 0.3 | | | | 1.00 | 92.4 (85.9, 99.3) | | | -0.3 | 1.00 |  |  |
| Subtype | 1.6 (1.4, 1.8) | | 0.0 | | | 1.00 | 4.4 (4.1, 4.8) | | | 0.0 | 1.00 | 22.7 (21.9, 23.6) | | | 0.0 | 1.00 | | 74.2 (72.2, 76.3) | | 0.0 | | | | 1.00 | 92.7 (86.2, 99.7) | | | 0.0 | 1.00 |  |  |
| xNMSC | 1.5 (1.4, 1.8) | | 0.0 | | | 0.99 | 4.3 (4.0, 4.7) | | | -0.1 | 0.98 | 22.1 (21.3, 22.9) | | | -0.7 | 0.97 | | 71.8 (69.7, 74.0) | | -2.4 | | | | 0.97 | 92.7 (85.4, 100.5) | | | 0.0 | 1.00 |  |  |
| First ever | 1.5 (1.4, 1.8) | | 0.0 | | | 0.99 | 4.3 (4.0, 4.7) | | | -0.1 | 0.98 | 22.0 (21.2, 22.8) | | | -0.8 | 0.97 | | 70.9 (68.8, 73.1) | | -3.3 | | | | 0.96 | 91.2 (83.8, 99.2) | | | -1.5 | 0.98 |  |  |
| C88 & C90 **Multiple myeloma** | | | | | |  |  | | |  |  |  | | |  |  | |  | |  | | | |  |  | | |  |  |  |  |
| Aggr | 0.0 (0.0, 0.0) | |  | | |  | 0.7 (0.6, 0.8) | | | 0.0 | 0.99 | 8.0 (7.5, 8.5) | | | 0.1 | 1.01 | | 31.9 (30.6, 33.3) | | 0.0 | | | | 1.00 | 34.9 (31.0, 39.3) | | | 0.3 | 1.01 |  |  |
| Subtype | 0.0 (0.0, 0.0) | |  | | |  | 0.7 (0.6, 0.9) | | | 0.0 | 1.00 | 7.9 (7.4, 8.4) | | | 0.0 | 1.00 | | 31.9 (30.6, 33.3) | | 0.0 | | | | 1.00 | 34.6 (30.7, 39.0) | | | 0.0 | 1.00 |  |  |
| xNMSC | 0.0 (0.0, 0.0) | |  | | |  | 0.7 (0.6, 0.9) | | | 0.0 | 1.01 | 7.6 (7.1, 8.1) | | | -0.3 | 0.96 | | 30.6 (29.2, 32.0) | | -1.3 | | | | 0.96 | 35.3 (30.9, 40.3) | | | 0.7 | 1.02 |  |  |
| First ever | 0.0 (0.0, 0.0) | |  | | |  | 0.7 (0.6, 0.9) | | | 0.0 | 1.01 | 7.6 (7.1, 8.1) | | | -0.3 | 0.96 | | 30.6 (29.2, 32.0) | | -1.3 | | | | 0.96 | 34.6 (30.2, 39.6) | | | -0.1 | 1.00 |  |  |
| C91-C95 **Leukemia** | | |  | | |  |  | | |  |  |  | | |  |  | |  | |  | | | |  |  | | |  |  |  |  |
| Aggr | 4.0 (3.7, 4.4) | | 0.1 | | | 1.01 | 3.0 (2.8, 3.3) | | | 0.0 | 1.01 | 14.6 (14.0, 15.3) | | | 0.1 | 1.01 | | 55.6 (53.9, 57.5) | | 0.6 | | | | 1.01 | 63.8 (58.4, 69.6) | | | 0.2 | 1.00 |  |  |
| Subtype | 4.0 (3.7, 4.3) | | 0.0 | | | 1.00 | 3.0 (2.7, 3.3) | | | 0.0 | 1.00 | 14.5 (13.9, 15.2) | | | 0.0 | 1.00 | | 55.1 (53.3, 56.9) | | 0.0 | | | | 1.00 | 63.5 (58.2, 69.3) | | | 0.0 | 1.00 |  |  |
| xNMSC | 3.9 (3.6, 4.3) | | 0.0 | | | 0.99 | 2.9 (2.6, 3.2) | | | -0.1 | 0.96 | 13.8 (13.1, 14.4) | | | -0.8 | 0.95 | | 52.2 (50.4, 54.1) | | -2.8 | | | | 0.95 | 62.7 (56.8, 69.2) | | | -0.8 | 0.99 |  |  |
| First ever | 3.9 (3.6, 4.3) | | 0.0 | | | 0.99 | 2.9 (2.6, 3.2) | | | -0.1 | 0.96 | 13.8 (13.1, 14.4) | | | -0.8 | 0.95 | | 51.6 (49.8, 53.5) | | -3.4 | | | | 0.94 | 60.6 (54.7, 67.2) | | | -2.9 | 0.95 |  |  |

**Supplementary Table 3:** Age-group-specific incidence rates (IR) (95% confidence intervals [95% CI]) per 100,000 person-years for 27 cancer subtypes in Swedish females between 2000 and 2010. IRs were calculated using four definitions of the study population: persons resident in Sweden 1) based on aggregate general population statistics (Aggr); 2) with no previous subtype-specific cancer diagnosis (subtype); 3) with no previous cancer diagnosis except non-melanoma skin cancer (xNMSC); and 4) with no previous cancer diagnosis of any type (First ever). IRs are presented with incidence rate differences (IRD) and incidence rate ratios (IRR) compared to subtype-specific IRs (ref).

| **Legend** | | |  | |  | | |  |  | | | |  |  | | | |  | |  | |  |  | | | |  |  | | |
| --- | --- | --- | --- | --- | --- | --- | --- | --- | --- | --- | --- | --- | --- | --- | --- | --- | --- | --- | --- | --- | --- | --- | --- | --- | --- | --- | --- | --- | --- | --- |
| Incidence rate difference (IRD) | | |  | | ≥10 higher than ref | | |  | ≥5 higher than ref | | | |  | <5 higher than ref | | | |  | | <5 lower than ref | |  | ≥5 lower than ref | | | |  | ≥10 lower than ref | | |
| Incidence rate ratio (IRR) | | |  | | ≥10% higher than ref | | |  | ≥5% higher than ref | | | |  | <5% higher than ref | | | |  | | <5% lower than ref | |  | ≥5% lower than ref | | | |  | ≥10% lower than ref | | |
|  | |  | | | |  |  | | | |  |  | | | | |  | |  | | | | |  |  | | | | |  |
| **Females** | | **0–24 years** | | | |  | **25–44 years** | | | |  | **45–64 years** | | | | |  | | **65–84 years** | | | | |  | **85+ years** | | | | |  |
|  | | **IR (95% CI)** | | **IRD** | | **IRR** | **IR (95% CI)** | | | **IRD** | **IRR** | **IR (95% CI)** | | | **IRD** | | **IRR** | | **IR (95% CI)** | | **IRD** | | | **IRR** | **IR (95% CI)** | | | | **IRD** | **IRR** |
| C00-C14 **Lip, oral cavity, and pharynx** | | | | |  |  | | |  |  |  | | | |  |  | |  | |  | | |  | |  | | |  |  |  |
| Aggr | 0.3 (0.2, 0.4) | | 0.0 | | 1.02 | 1.8 (1.6, 2.0) | | | 0.0 | 1.03 | 9.5 (9.0, 10.1) | | | | 0.3 | 1.04 | | 21.7 (20.7, 22.7) | | 1.3 | | | 1.06 | | 30.6 (28.1, 33.4) | | | 2.1 | 1.07 |  |
| Subtype | 0.3 (0.2, 0.4) | | 0.0 | | 1.00 | 1.7 (1.5, 2.0) | | | 0.0 | 1.00 | 9.2 (8.7, 9.7) | | | | 0.0 | 1.00 | | 20.4 (19.4, 21.4) | | 0.0 | | | 1.00 | | 28.5 (26.1, 31.2) | | | 0.0 | 1.00 |  |
| xNMSC | 0.3 (0.2, 0.4) | | 0.0 | | 1.00 | 1.7 (1.5, 1.9) | | | -0.1 | 0.97 | 8.8 (8.3, 9.3) | | | | -0.4 | 0.96 | | 19.5 (18.5, 20.6) | | -0.9 | | | 0.96 | | 28.4 (25.7, 31.3) | | | -0.2 | 0.99 |  |
| First ever | 0.3 (0.2, 0.4) | | 0.0 | | 1.00 | 1.6 (1.4, 1.9) | | | -0.1 | 0.95 | 8.8 (8.3, 9.3) | | | | -0.4 | 0.96 | | 19.2 (18.2, 20.3) | | -1.2 | | | 0.94 | | 27.0 (24.4, 29.9) | | | -1.5 | 0.95 |  |
| C11 **Nasopharynx** | | |  | |  |  | | |  |  |  | | | |  |  | |  | |  | | |  | |  | | |  |  |  |
| Aggr | 0.1 (0.0, 0.1) | | 0.0 | | 1.00 | 0.1 (0.1, 0.2) | | | 0.0 | 0.99 | 0.3 (0.2, 0.4) | | | | 0.0 | 1.02 | | 0.5 (0.4, 0.7) | | 0.0 | | | 1.00 | | 0.2 (0.1, 0.6) | | | 0.0 | 1.00 |  |
| Subtype | 0.1 (0.0, 0.1) | | 0.0 | | 1.00 | 0.1 (0.1, 0.2) | | | 0.0 | 1.00 | 0.3 (0.2, 0.4) | | | | 0.0 | 1.00 | | 0.5 (0.4, 0.7) | | 0.0 | | | 1.00 | | 0.2 (0.1, 0.6) | | | 0.0 | 1.00 |  |
| xNMSC | 0.1 (0.0, 0.1) | | 0.0 | | 1.00 | 0.1 (0.1, 0.2) | | | 0.0 | 1.01 | 0.3 (0.2, 0.4) | | | | 0.0 | 0.95 | | 0.5 (0.3, 0.7) | | 0.0 | | | 0.98 | | 0.3 (0.1, 0.7) | | | 0.0 | 1.18 |  |
| First ever | 0.1 (0.0, 0.1) | | 0.0 | | 1.00 | 0.1 (0.1, 0.2) | | | 0.0 | 0.94 | 0.3 (0.2, 0.4) | | | | 0.0 | 0.95 | | 0.5 (0.3, 0.7) | | 0.0 | | | 0.98 | | 0.3 (0.1, 0.8) | | | 0.0 | 1.21 |  |
| C15 **Esophagus** | | |  | |  |  | | |  |  |  | | | |  |  | |  | |  | | |  | |  | | |  |  |  |
| Aggr | 0.0 (0.0, 0.0) | |  | |  | 0.1 (0.1, 0.2) | | | 0.0 | 0.99 | 2.3 (2.1, 2.6) | | | | 0.0 | 1.00 | | 8.7 (8.1, 9.4) | | 0.0 | | | 1.00 | | 11.8 (10.3, 13.6) | | | 0.0 | 1.00 |  |
| Subtype | 0.0 (0.0, 0.0) | |  | |  | 0.1 (0.1, 0.2) | | | 0.0 | 1.00 | 2.3 (2.1, 2.6) | | | | 0.0 | 1.00 | | 8.7 (8.1, 9.4) | | 0.0 | | | 1.00 | | 11.8 (10.2, 13.5) | | | 0.0 | 1.00 |  |
| xNMSC | 0.0 (0.0, 0.0) | |  | |  | 0.1 (0.1, 0.2) | | | 0.0 | 0.85 | 2.1 (1.9, 2.4) | | | | -0.2 | 0.91 | | 8.3 (7.7, 9.0) | | -0.4 | | | 0.96 | | 11.5 (9.8, 13.4) | | | -0.3 | 0.98 |  |
| First ever | 0.0 (0.0, 0.0) | |  | |  | 0.1 (0.1, 0.2) | | | 0.0 | 0.85 | 2.1 (1.9, 2.4) | | | | -0.2 | 0.91 | | 8.3 (7.6, 9.0) | | -0.5 | | | 0.95 | | 11.4 (9.8, 13.3) | | | -0.3 | 0.97 |  |
| C16 **Stomach** | | |  | |  |  | | |  |  |  | | | |  |  | |  | |  | | |  | |  | | |  |  |  |
| Aggr | 0.0 (0.0, 0.1) | | 0.0 | | 1.16 | 1.1 (0.9, 1.3) | | | 0.0 | 1.00 | 6.9 (6.5, 7.4) | | | | 0.0 | 1.00 | | 29.7 (28.5, 30.9) | | 0.1 | | | 1.00 | | 40.6 (37.7, 43.8) | | | 0.2 | 1.01 |  |
| Subtype | 0.0 (0.0, 0.1) | | 0.0 | | 1.00 | 1.1 (0.9, 1.3) | | | 0.0 | 1.00 | 6.9 (6.5, 7.4) | | | | 0.0 | 1.00 | | 29.5 (28.4, 30.7) | | 0.0 | | | 1.00 | | 40.4 (37.5, 43.6) | | | 0.0 | 1.00 |  |
| xNMSC | 0.0 (0.0, 0.1) | | 0.0 | | 1.00 | 1.1 (0.9, 1.3) | | | 0.0 | 0.98 | 6.6 (6.1, 7.1) | | | | -0.3 | 0.95 | | 28.0 (26.8, 29.3) | | -1.6 | | | 0.95 | | 39.4 (36.3, 42.8) | | | -1.0 | 0.98 |  |
| First ever | 0.0 (0.0, 0.1) | | 0.0 | | 1.00 | 1.1 (0.9, 1.3) | | | 0.0 | 0.98 | 6.6 (6.2, 7.1) | | | | -0.3 | 0.95 | | 27.9 (26.7, 29.2) | | -1.7 | | | 0.94 | | 39.1 (35.9, 42.5) | | | -1.3 | 0.97 |  |
| C17-C21 **Intestine** | | |  | |  |  | | |  |  |  | | | |  |  | |  | |  | | |  | |  | | |  |  |  |
| Aggr | 0.8 (0.6, 0.9) | | 0.0 | | 1.03 | 6.7 (6.2, 7.1) | | | 0.2 | 1.03 | 56.2 (54.9, 57.5) | | | | 1.6 | 1.03 | | 239.9 (236.6, 243.3) | | 11.4 | | | 1.05 | | 271.7 (263.9, 279.6) | | | 11.6 | 1.04 |  |
| Subtype | 0.8 (0.6, 0.9) | | 0.0 | | 1.00 | 6.5 (6.1, 6.9) | | | 0.0 | 1.00 | 54.5 (53.2, 55.8) | | | | 0.0 | 1.00 | | 228.6 (225.3, 231.9) | | 0.0 | | | 1.00 | | 260.1 (252.4, 268.0) | | | 0.0 | 1.00 |  |
| xNMSC | 0.7 (0.6, 0.9) | | 0.0 | | 0.98 | 6.4 (5.9, 6.8) | | | -0.1 | 0.98 | 52.3 (51.0, 53.6) | | | | -2.2 | 0.96 | | 221.0 (217.6, 224.5) | | -7.5 | | | 0.97 | | 254.3 (246.1, 262.7) | | | -5.8 | 0.98 |  |
| First ever | 0.7 (0.6, 0.9) | | 0.0 | | 0.98 | 6.4 (5.9, 6.8) | | | -0.1 | 0.98 | 52.3 (51.0, 53.6) | | | | -2.2 | 0.96 | | 220.6 (217.2, 224.1) | | -8.0 | | | 0.97 | | 253.1 (244.9, 261.6) | | | -7.0 | 0.97 |  |
| C18 **Colon** |  | |  | |  |  | | |  |  |  | | | |  |  | |  | |  | | |  | |  | | |  |  |  |
| Aggr | 0.7 (0.6, 0.9) | | 0.0 | | 1.01 | 4.0 (3.6, 4.3) | | | 0.1 | 1.02 | 32.3 (31.3, 33.3) | | | | 0.9 | 1.03 | | 163.9 (161.2, 166.7) | | 8.2 | | | 1.05 | | 189.5 (183.1, 196.2) | | | 7.7 | 1.04 |  |
| Subtype | 0.7 (0.6, 0.9) | | 0.0 | | 1.00 | 3.9 (3.6, 4.2) | | | 0.0 | 1.00 | 31.4 (30.5, 32.4) | | | | 0.0 | 1.00 | | 155.7 (153.0, 158.5) | | 0.0 | | | 1.00 | | 181.9 (175.5, 188.5) | | | 0.0 | 1.00 |  |
| xNMSC | 0.7 (0.6, 0.8) | | 0.0 | | 0.98 | 3.8 (3.5, 4.1) | | | -0.1 | 0.98 | 29.9 (29.0, 30.9) | | | | -1.5 | 0.95 | | 149.6 (146.8, 152.5) | | -6.2 | | | 0.96 | | 174.7 (168.0, 181.7) | | | -7.2 | 0.96 |  |
| First ever | 0.7 (0.6, 0.8) | | 0.0 | | 0.98 | 3.8 (3.5, 4.1) | | | -0.1 | 0.98 | 29.9 (29.0, 30.9) | | | | -1.5 | 0.95 | | 149.3 (146.4, 152.1) | | -6.5 | | | 0.96 | | 173.6 (166.8, 180.7) | | | -8.3 | 0.95 |  |
| C18-C21 **Colon, rectum, and anus** | | | | |  |  | | |  |  |  | | | |  |  | |  | |  | | |  | |  | | |  |  |  |
| Aggr | 0.8 (0.6, 0.9) | | 0.0 | | 1.04 | 6.3 (5.9, 6.7) | | | 0.2 | 1.03 | 53.4 (52.2, 54.7) | | | | 1.4 | 1.03 | | 231.6 (228.3, 234.9) | | 10.3 | | | 1.05 | | 265.0 (257.4, 272.9) | | | 10.8 | 1.04 |  |
| Subtype | 0.7 (0.6, 0.9) | | 0.0 | | 1.00 | 6.1 (5.7, 6.5) | | | 0.0 | 1.00 | 52.0 (50.8, 53.3) | | | | 0.0 | 1.00 | | 221.3 (218.1, 224.6) | | 0.0 | | | 1.00 | | 254.3 (246.6, 262.1) | | | 0.0 | 1.00 |  |
| xNMSC | 0.7 (0.6, 0.9) | | 0.0 | | 0.98 | 6.0 (5.6, 6.4) | | | -0.1 | 0.98 | 49.9 (48.7, 51.2) | | | | -2.1 | 0.96 | | 214.1 (210.8, 217.6) | | -7.2 | | | 0.97 | | 247.7 (239.7, 256.1) | | | -6.5 | 0.97 |  |
| First ever | 0.7 (0.6, 0.9) | | 0.0 | | 0.98 | 6.0 (5.6, 6.4) | | | -0.1 | 0.98 | 49.9 (48.6, 51.1) | | | | -2.2 | 0.96 | | 213.7 (210.3, 217.1) | | -7.6 | | | 0.97 | | 246.6 (238.5, 255.0) | | | -7.7 | 0.97 |  |
| C19-C21 **Rectum and anus** | | | | |  |  | | |  |  |  | | | |  |  | |  | |  | | |  | |  | | |  |  |  |
| Aggr | 0.1 (0.0, 0.1) | | 0.0 | | 1.14 | 2.3 (2.1, 2.6) | | | 0.0 | 1.02 | 21.2 (20.4, 22.0) | | | | 0.1 | 1.00 | | 67.7 (65.9, 69.5) | | 0.1 | | | 1.00 | | 75.5 (71.4, 79.7) | | | 0.6 | 1.01 |  |
| Subtype | 0.0 (0.0, 0.1) | | 0.0 | | 1.00 | 2.3 (2.0, 2.5) | | | 0.0 | 1.00 | 21.1 (20.3, 21.9) | | | | 0.0 | 1.00 | | 67.6 (65.8, 69.4) | | 0.0 | | | 1.00 | | 74.9 (70.8, 79.1) | | | 0.0 | 1.00 |  |
| xNMSC | 0.0 (0.0, 0.1) | | 0.0 | | 0.72 | 2.2 (2.0, 2.5) | | | 0.0 | 0.98 | 20.1 (19.3, 20.9) | | | | -1.0 | 0.95 | | 65.1 (63.3, 67.0) | | -2.4 | | | 0.96 | | 73.7 (69.4, 78.3) | | | -1.1 | 0.98 |  |
| First ever | 0.0 (0.0, 0.1) | | 0.0 | | 0.72 | 2.2 (2.0, 2.5) | | | 0.0 | 0.98 | 20.1 (19.3, 20.9) | | | | -1.0 | 0.95 | | 65.0 (63.1, 66.9) | | -2.6 | | | 0.96 | | 73.7 (69.3, 78.3) | | | -1.2 | 0.98 |  |
| C22 **Liver (specified as primary)** | | | | |  |  | | |  |  |  | | | |  |  | |  | |  | | |  | |  | | |  |  |  |
| Aggr | 0.2 (0.1, 0.2) | | 0.0 | | 1.10 | 0.4 (0.3, 0.5) | | | 0.0 | 1.01 | 3.0 (2.8, 3.4) | | | | 0.0 | 1.00 | | 11.1 (10.4, 11.8) | | 0.1 | | | 1.01 | | 7.5 (6.3, 8.9) | | | -0.1 | 0.98 |  |
| Subtype | 0.1 (0.1, 0.2) | | 0.0 | | 1.00 | 0.4 (0.3, 0.5) | | | 0.0 | 1.00 | 3.0 (2.7, 3.3) | | | | 0.0 | 1.00 | | 11.0 (10.3, 11.8) | | 0.0 | | | 1.00 | | 7.6 (6.4, 9.0) | | | 0.0 | 1.00 |  |
| xNMSC | 0.1 (0.1, 0.2) | | 0.0 | | 1.00 | 0.4 (0.3, 0.5) | | | 0.0 | 1.01 | 2.9 (2.6, 3.2) | | | | -0.1 | 0.96 | | 10.8 (10.0, 11.6) | | -0.3 | | | 0.98 | | 8.0 (6.7, 9.6) | | | 0.4 | 1.06 |  |
| First ever | 0.1 (0.1, 0.2) | | 0.0 | | 1.00 | 0.4 (0.3, 0.5) | | | 0.0 | 1.01 | 2.9 (2.6, 3.2) | | | | -0.1 | 0.95 | | 10.8 (10.0, 11.6) | | -0.3 | | | 0.98 | | 8.2 (6.8, 9.8) | | | 0.6 | 1.08 |  |
| C23-C24 **Gallbladder** | | | | |  |  | | |  |  |  | | | |  |  | |  | |  | | |  | |  | | |  |  |  |
| Aggr | 0.0 (0.0, 0.0) | |  | |  | 0.1 (0.1, 0.2) | | | 0.0 | 0.91 | 2.7 (2.4, 3.0) | | | | 0.0 | 1.00 | | 12.2 (11.5, 13.0) | | 0.0 | | | 1.00 | | 9.6 (8.2, 11.1) | | | 0.0 | 1.00 |  |
| Subtype | 0.0 (0.0, 0.0) | |  | |  | 0.1 (0.1, 0.2) | | | 0.0 | 1.00 | 2.7 (2.4, 3.0) | | | | 0.0 | 1.00 | | 12.2 (11.5, 13.0) | | 0.0 | | | 1.00 | | 9.6 (8.2, 11.2) | | | 0.0 | 1.00 |  |
| xNMSC | 0.0 (0.0, 0.0) | |  | |  | 0.1 (0.1, 0.2) | | | 0.0 | 1.01 | 2.6 (2.4, 3.0) | | | | -0.1 | 0.97 | | 12.4 (11.6, 13.2) | | 0.2 | | | 1.01 | | 10.1 (8.5, 11.9) | | | 0.5 | 1.05 |  |
| First ever | 0.0 (0.0, 0.0) | |  | |  | 0.1 (0.1, 0.2) | | | 0.0 | 1.01 | 2.6 (2.4, 2.9) | | | | -0.1 | 0.97 | | 12.4 (11.6, 13.2) | | 0.2 | | | 1.01 | | 9.8 (8.3, 11.6) | | | 0.3 | 1.03 |  |
| C25 **Pancreas** | | |  | |  |  | | |  |  |  | | | |  |  | |  | |  | | |  | |  | | |  |  |  |
| Aggr | 0.0 (0.0, 0.1) | | 0.0 | | 1.00 | 0.6 (0.5, 0.8) | | | 0.0 | 1.01 | 10.9 (10.3, 11.5) | | | | 0.0 | 1.00 | | 38.4 (37.1, 39.7) | | 0.0 | | | 1.00 | | 26.2 (23.8, 28.7) | | | 0.0 | 1.00 |  |
| Subtype | 0.0 (0.0, 0.1) | | 0.0 | | 1.00 | 0.6 (0.5, 0.7) | | | 0.0 | 1.00 | 10.9 (10.3, 11.5) | | | | 0.0 | 1.00 | | 38.4 (37.1, 39.8) | | 0.0 | | | 1.00 | | 26.1 (23.8, 28.7) | | | 0.0 | 1.00 |  |
| xNMSC | 0.0 (0.0, 0.1) | | 0.0 | | 1.00 | 0.6 (0.5, 0.7) | | | 0.0 | 0.98 | 10.4 (9.8, 11.0) | | | | -0.5 | 0.96 | | 37.5 (36.1, 38.9) | | -0.9 | | | 0.98 | | 26.5 (23.9, 29.3) | | | 0.3 | 1.01 |  |
| First ever | 0.0 (0.0, 0.1) | | 0.0 | | 1.00 | 0.6 (0.5, 0.7) | | | 0.0 | 0.98 | 10.4 (9.8, 11.0) | | | | -0.5 | 0.95 | | 37.5 (36.1, 38.9) | | -0.9 | | | 0.98 | | 26.3 (23.7, 29.1) | | | 0.2 | 1.01 |  |
| C32 **Larynx** |  | |  | |  |  | | |  |  |  | | | |  |  | |  | |  | | |  | |  | | |  |  |  |
| Aggr | 0.0 (0.0, 0.0) | |  | |  | 0.1 (0.1, 0.2) | | | 0.0 | 0.99 | 1.0 (0.8, 1.2) | | | | 0.0 | 1.00 | | 2.2 (1.9, 2.5) | | 0.0 | | | 1.00 | | 1.5 (1.0, 2.2) | | | 0.0 | 1.00 |  |
| Subtype | 0.0 (0.0, 0.0) | |  | |  | 0.1 (0.1, 0.2) | | | 0.0 | 1.00 | 1.0 (0.8, 1.2) | | | | 0.0 | 1.00 | | 2.2 (1.9, 2.5) | | 0.0 | | | 1.00 | | 1.5 (1.0, 2.2) | | | 0.0 | 1.00 |  |
| xNMSC | 0.0 (0.0, 0.0) | |  | |  | 0.1 (0.1, 0.2) | | | 0.0 | 1.01 | 1.0 (0.8, 1.2) | | | | 0.0 | 0.99 | | 2.0 (1.7, 2.4) | | -0.1 | | | 0.94 | | 1.4 (0.9, 2.2) | | | -0.1 | 0.95 |  |
| First ever | 0.0 (0.0, 0.0) | |  | |  | 0.1 (0.1, 0.2) | | | 0.0 | 1.01 | 1.0 (0.8, 1.2) | | | | 0.0 | 0.99 | | 2.0 (1.7, 2.4) | | -0.1 | | | 0.94 | | 1.4 (0.9, 2.1) | | | -0.1 | 0.92 |  |
| C33-C34 **Lung (incl. trachea and bronchus)** | | | | | |  | | |  |  |  | | | |  |  | |  | |  | | |  | |  | | |  |  |  |
| Aggr | 0.1 (0.1, 0.2) | | 0.0 | | 1.00 | 2.2 (1.9, 2.5) | | | 0.0 | 1.00 | 48.9 (47.7, 50.1) | | | | 0.6 | 1.01 | | 125.0 (122.6, 127.5) | | 1.7 | | | 1.01 | | 49.2 (45.9, 52.6) | | | 0.3 | 1.01 |  |
| Subtype | 0.1 (0.1, 0.2) | | 0.0 | | 1.00 | 2.2 (1.9, 2.5) | | | 0.0 | 1.00 | 48.2 (47.0, 49.5) | | | | 0.0 | 1.00 | | 123.3 (120.9, 125.8) | | 0.0 | | | 1.00 | | 48.9 (45.6, 52.3) | | | 0.0 | 1.00 |  |
| xNMSC | 0.1 (0.1, 0.2) | | 0.0 | | 1.00 | 2.1 (1.9, 2.4) | | | -0.1 | 0.96 | 45.7 (44.5, 46.9) | | | | -2.5 | 0.95 | | 117.1 (114.6, 119.7) | | -6.2 | | | 0.95 | | 47.2 (43.8, 50.9) | | | -1.7 | 0.97 |  |
| First ever | 0.1 (0.1, 0.2) | | 0.0 | | 1.00 | 2.1 (1.9, 2.4) | | | -0.1 | 0.96 | 45.6 (44.4, 46.9) | | | | -2.6 | 0.95 | | 117.0 (114.5, 119.5) | | -6.3 | | | 0.95 | | 47.0 (43.6, 50.8) | | | -1.8 | 0.96 |  |
| C43 **Melanoma of skin** | | | | |  |  | | |  |  |  | | | |  |  | |  | |  | | |  | |  | | |  |  |  |
| Aggr | 1.5 (1.3, 1.7) | | 0.0 | | 1.02 | 18.0 (17.3, 18.7) | | | 0.6 | 1.03 | 33.8 (32.8, 34.8) | | | | 1.1 | 1.03 | | 52.0 (50.5, 53.6) | | 3.1 | | | 1.06 | | 62.7 (59.0, 66.6) | | | 4.4 | 1.08 |  |
| Subtype | 1.5 (1.3, 1.7) | | 0.0 | | 1.00 | 17.4 (16.7, 18.1) | | | 0.0 | 1.00 | 32.7 (31.7, 33.7) | | | | 0.0 | 1.00 | | 48.9 (47.4, 50.4) | | 0.0 | | | 1.00 | | 58.3 (54.7, 62.0) | | | 0.0 | 1.00 |  |
| xNMSC | 1.5 (1.3, 1.7) | | 0.0 | | 1.00 | 17.2 (16.5, 18.0) | | | -0.2 | 0.99 | 32.1 (31.1, 33.1) | | | | -0.6 | 0.98 | | 47.4 (45.9, 49.1) | | -1.4 | | | 0.97 | | 56.2 (52.5, 60.3) | | | -2.0 | 0.96 |  |
| First ever | 1.5 (1.3, 1.7) | | 0.0 | | 1.00 | 17.2 (16.5, 18.0) | | | -0.2 | 0.99 | 32.0 (31.0, 33.0) | | | | -0.7 | 0.98 | | 47.1 (45.5, 48.7) | | -1.8 | | | 0.96 | | 54.7 (51.0, 58.8) | | | -3.5 | 0.94 |  |
| C50 **Breast** |  | |  | |  |  | | |  |  |  | | | |  |  | |  | |  | | |  | |  | | |  |  |  |
| Aggr | 0.2 (0.1, 0.2) | | 0.0 | | 1.00 | 51.1 (49.9, 52.3) | | | 3.5 | 1.07 | 277.9 (275.1, 280.8) | | | | 19.2 | 1.07 | | 360.1 (356.0, 364.2) | | 29.6 | | | 1.09 | | 343.1 (334.4, 352.0) | | | 21.1 | 1.07 |  |
| Subtype | 0.2 (0.1, 0.2) | | 0.0 | | 1.00 | 47.6 (46.4, 48.8) | | | 0.0 | 1.00 | 258.7 (255.9, 261.6) | | | | 0.0 | 1.00 | | 330.5 (326.5, 334.6) | | 0.0 | | | 1.00 | | 322.0 (313.3, 331.0) | | | 0.0 | 1.00 |  |
| xNMSC | 0.2 (0.1, 0.2) | | 0.0 | | 1.00 | 47.1 (46.0, 48.3) | | | -0.5 | 0.99 | 256.8 (254.0, 259.7) | | | | -1.9 | 0.99 | | 326.2 (322.0, 330.4) | | -4.3 | | | 0.99 | | 315.5 (306.4, 324.9) | | | -6.5 | 0.98 |  |
| First ever | 0.2 (0.1, 0.2) | | 0.0 | | 1.00 | 47.1 (46.0, 48.3) | | | -0.5 | 0.99 | 256.7 (253.8, 259.6) | | | | -2.1 | 0.99 | | 325.7 (321.5, 329.9) | | -4.8 | | | 0.99 | | 314.4 (305.2, 323.8) | | | -7.6 | 0.98 |  |
| C53 **Cervix uteri** | | |  | |  |  | | |  |  |  | | | |  |  | |  | |  | | |  | |  | | |  |  |  |
| Aggr | 0.4 (0.3, 0.5) | | 0.0 | | 1.02 | 14.1 (13.4, 14.7) | | | 0.1 | 1.01 | 12.0 (11.4, 12.6) | | | | 0.0 | 1.00 | | 15.1 (14.3, 16.0) | | 0.1 | | | 1.00 | | 14.9 (13.2, 16.9) | | | -0.1 | 0.99 |  |
| Subtype | 0.4 (0.3, 0.5) | | 0.0 | | 1.00 | 14.0 (13.3, 14.6) | | | 0.0 | 1.00 | 12.0 (11.4, 12.6) | | | | 0.0 | 1.00 | | 15.1 (14.2, 15.9) | | 0.0 | | | 1.00 | | 15.0 (13.2, 16.9) | | | 0.0 | 1.00 |  |
| xNMSC | 0.4 (0.3, 0.5) | | 0.0 | | 1.00 | 14.0 (13.3, 14.6) | | | 0.0 | 1.00 | 11.9 (11.3, 12.6) | | | | 0.0 | 1.00 | | 15.1 (14.2, 16.1) | | 0.1 | | | 1.00 | | 15.4 (13.5, 17.6) | | | 0.4 | 1.03 |  |
| First ever | 0.4 (0.3, 0.5) | | 0.0 | | 1.00 | 14.0 (13.3, 14.6) | | | 0.0 | 1.00 | 11.9 (11.3, 12.6) | | | | 0.0 | 1.00 | | 15.2 (14.3, 16.1) | | 0.1 | | | 1.01 | | 15.7 (13.7, 17.9) | | | 0.7 | 1.05 |  |
| C53-C55 **Uterus** | | |  | |  |  | | |  |  |  | | | |  |  | |  | |  | | |  | |  | | |  |  |  |
| Aggr | 0.4 (0.3, 0.5) | | 0.0 | | 1.01 | 16.3 (15.6, 17.0) | | | 0.1 | 1.01 | 55.3 (54.0, 56.6) | | | | 0.0 | 1.00 | | 123.8 (121.4, 126.2) | | -2.0 | | | 0.98 | | 91.1 (86.6, 95.7) | | | -1.9 | 0.98 |  |
| Subtype | 0.4 (0.3, 0.5) | | 0.0 | | 1.00 | 16.2 (15.5, 16.9) | | | 0.0 | 1.00 | 55.3 (54.0, 56.6) | | | | 0.0 | 1.00 | | 125.8 (123.4, 128.3) | | 0.0 | | | 1.00 | | 92.9 (88.4, 97.7) | | | 0.0 | 1.00 |  |
| xNMSC | 0.4 (0.3, 0.5) | | 0.0 | | 1.00 | 16.1 (15.4, 16.8) | | | -0.1 | 0.99 | 54.0 (52.7, 55.3) | | | | -1.3 | 0.98 | | 123.6 (121.1, 126.2) | | -2.2 | | | 0.98 | | 89.2 (84.4, 94.3) | | | -3.7 | 0.96 |  |
| First ever | 0.4 (0.3, 0.5) | | 0.0 | | 1.00 | 16.1 (15.4, 16.8) | | | -0.1 | 0.99 | 54.0 (52.7, 55.3) | | | | -1.3 | 0.98 | | 123.5 (120.9, 126.1) | | -2.3 | | | 0.98 | | 88.8 (84.0, 93.9) | | | -4.2 | 0.96 |  |
| C54 **Corpus uteri** | | |  | |  |  | | |  |  |  | | | |  |  | |  | |  | | |  | |  | | |  |  |  |
| Aggr | 0.0 (0.0, 0.1) | | 0.0 | | 1.00 | 1.6 (1.4, 1.8) | | | 0.0 | 0.99 | 39.6 (38.5, 40.7) | | | | 0.0 | 1.00 | | 101.5 (99.3, 103.7) | | -1.7 | | | 0.98 | | 69.0 (65.1, 73.1) | | | -1.1 | 0.98 |  |
| Subtype | 0.0 (0.0, 0.1) | | 0.0 | | 1.00 | 1.6 (1.4, 1.8) | | | 0.0 | 1.00 | 39.6 (38.5, 40.7) | | | | 0.0 | 1.00 | | 103.1 (100.9, 105.4) | | 0.0 | | | 1.00 | | 70.1 (66.1, 74.2) | | | 0.0 | 1.00 |  |
| xNMSC | 0.0 (0.0, 0.1) | | 0.0 | | 1.00 | 1.5 (1.3, 1.7) | | | -0.1 | 0.94 | 38.4 (37.3, 39.6) | | | | -1.2 | 0.97 | | 101.7 (99.4, 104.1) | | -1.4 | | | 0.99 | | 66.8 (62.7, 71.2) | | | -3.3 | 0.95 |  |
| First ever | 0.0 (0.0, 0.1) | | 0.0 | | 1.00 | 1.5 (1.3, 1.7) | | | -0.1 | 0.94 | 38.4 (37.3, 39.5) | | | | -1.2 | 0.97 | | 101.5 (99.2, 103.9) | | -1.6 | | | 0.98 | | 66.3 (62.2, 70.7) | | | -3.8 | 0.95 |  |
| C56 **Ovary** |  | |  | |  |  | | |  |  |  | | | |  |  | |  | |  | | |  | |  | | |  |  |  |
| Aggr | 0.8 (0.6, 0.9) | | 0.0 | | 1.01 | 4.7 (4.3, 5.1) | | | 0.0 | 1.00 | 27.1 (26.2, 28.0) | | | | 0.1 | 1.00 | | 45.8 (44.4, 47.3) | | 0.0 | | | 1.00 | | 25.5 (23.2, 28.0) | | | 0.1 | 1.00 |  |
| Subtype | 0.8 (0.6, 0.9) | | 0.0 | | 1.00 | 4.7 (4.3, 5.1) | | | 0.0 | 1.00 | 26.9 (26.1, 27.9) | | | | 0.0 | 1.00 | | 45.8 (44.3, 47.3) | | 0.0 | | | 1.00 | | 25.4 (23.1, 28.0) | | | 0.0 | 1.00 |  |
| xNMSC | 0.8 (0.6, 0.9) | | 0.0 | | 0.99 | 4.6 (4.2, 5.0) | | | -0.1 | 0.97 | 25.8 (25.0, 26.8) | | | | -1.1 | 0.96 | | 45.8 (44.2, 47.4) | | 0.0 | | | 1.00 | | 26.0 (23.5, 28.8) | | | 0.6 | 1.02 |  |
| First ever | 0.8 (0.6, 0.9) | | 0.0 | | 0.99 | 4.6 (4.2, 5.0) | | | -0.1 | 0.97 | 25.8 (24.9, 26.8) | | | | -1.1 | 0.96 | | 45.7 (44.2, 47.3) | | -0.1 | | | 1.00 | | 25.9 (23.4, 28.7) | | | 0.5 | 1.02 |  |
| C64 **Kidney** |  | |  | |  |  | | |  |  |  | | | |  |  | |  | |  | | |  | |  | | |  |  |  |
| Aggr | 0.6 (0.5, 0.7) | | 0.0 | | 1.02 | 1.2 (1.1, 1.4) | | | 0.0 | 1.03 | 10.8 (10.2, 11.4) | | | | 0.2 | 1.02 | | 33.9 (32.6, 35.1) | | 0.6 | | | 1.02 | | 14.9 (13.2, 16.9) | | | 0.4 | 1.03 |  |
| Subtype | 0.6 (0.5, 0.7) | | 0.0 | | 1.00 | 1.2 (1.0, 1.4) | | | 0.0 | 1.00 | 10.6 (10.0, 11.2) | | | | 0.0 | 1.00 | | 33.2 (32.0, 34.5) | | 0.0 | | | 1.00 | | 14.5 (12.8, 16.4) | | | 0.0 | 1.00 |  |
| xNMSC | 0.6 (0.5, 0.7) | | 0.0 | | 1.00 | 1.1 (1.0, 1.3) | | | -0.1 | 0.94 | 9.7 (9.1, 10.3) | | | | -0.9 | 0.92 | | 30.2 (29.0, 31.6) | | -3.0 | | | 0.91 | | 13.2 (11.5, 15.3) | | | -1.2 | 0.91 |  |
| First ever | 0.6 (0.5, 0.7) | | 0.0 | | 1.00 | 1.1 (1.0, 1.3) | | | -0.1 | 0.94 | 9.7 (9.1, 10.2) | | | | -0.9 | 0.91 | | 30.3 (29.0, 31.6) | | -2.9 | | | 0.91 | | 13.3 (11.5, 15.3) | | | -1.2 | 0.92 |  |
| C67 **Bladder** |  | |  | |  |  | | |  |  |  | | | |  |  | |  | |  | | |  | |  | | |  |  |  |
| Aggr | 0.0 (0.0, 0.1) | | 0.0 | | 1.00 | 0.9 (0.7, 1.1) | | | 0.0 | 1.02 | 11.2 (10.6, 11.8) | | | | 0.1 | 1.01 | | 46.0 (44.5, 47.5) | | 0.2 | | | 1.00 | | 59.4 (55.9, 63.2) | | | 0.6 | 1.01 |  |
| Subtype | 0.0 (0.0, 0.1) | | 0.0 | | 1.00 | 0.9 (0.7, 1.0) | | | 0.0 | 1.00 | 11.1 (10.6, 11.7) | | | | 0.0 | 1.00 | | 45.8 (44.3, 47.3) | | 0.0 | | | 1.00 | | 58.9 (55.3, 62.7) | | | 0.0 | 1.00 |  |
| xNMSC | 0.0 (0.0, 0.1) | | 0.0 | | 1.00 | 0.8 (0.7, 1.0) | | | -0.1 | 0.94 | 10.3 (9.8, 10.9) | | | | -0.8 | 0.93 | | 40.7 (39.2, 42.2) | | -5.1 | | | 0.89 | | 52.3 (48.7, 56.2) | | | -6.5 | 0.89 |  |
| First ever | 0.0 (0.0, 0.1) | | 0.0 | | 1.00 | 0.8 (0.7, 1.0) | | | -0.1 | 0.94 | 10.3 (9.8, 10.9) | | | | -0.8 | 0.93 | | 40.5 (39.0, 42.0) | | -5.3 | | | 0.88 | | 52.6 (48.9, 56.5) | | | -6.3 | 0.89 |  |
| C70-C72 **Brain and central nervous system** | | | | | |  | | |  |  |  | | | |  |  | |  | |  | | |  | |  | | |  |  |  |
| Aggr | 4.1 (3.8, 4.4) | | 0.1 | | 1.03 | 8.8 (8.3, 9.3) | | | 0.3 | 1.03 | 25.8 (24.9, 26.7) | | | | 0.4 | 1.01 | | 30.0 (28.8, 31.2) | | 0.3 | | | 1.01 | | 11.7 (10.2, 13.4) | | | 0.6 | 1.05 |  |
| Subtype | 4.0 (3.7, 4.3) | | 0.0 | | 1.00 | 8.5 (8.0, 9.1) | | | 0.0 | 1.00 | 25.4 (24.6, 26.3) | | | | 0.0 | 1.00 | | 29.7 (28.5, 30.9) | | 0.0 | | | 1.00 | | 11.1 (9.6, 12.8) | | | 0.0 | 1.00 |  |
| xNMSC | 4.0 (3.6, 4.3) | | 0.0 | | 0.99 | 8.4 (7.9, 8.9) | | | -0.1 | 0.99 | 25.0 (24.1, 25.9) | | | | -0.5 | 0.98 | | 29.1 (27.9, 30.4) | | -0.6 | | | 0.98 | | 10.7 (9.1, 12.5) | | | -0.4 | 0.97 |  |
| First ever | 4.0 (3.6, 4.3) | | 0.0 | | 0.99 | 8.4 (7.9, 8.9) | | | -0.1 | 0.99 | 24.9 (24.1, 25.8) | | | | -0.5 | 0.98 | | 29.2 (27.9, 30.5) | | -0.5 | | | 0.98 | | 10.7 (9.1, 12.6) | | | -0.4 | 0.97 |  |
| C73 **Thyroid** | | |  | |  |  | | |  |  |  | | | |  |  | |  | |  | | |  | |  | | |  |  |  |
| Aggr | 1.3 (1.1, 1.5) | | 0.0 | | 1.04 | 6.1 (5.7, 6.6) | | | 0.1 | 1.01 | 6.9 (6.5, 7.4) | | | | 0.1 | 1.01 | | 8.3 (7.7, 8.9) | | 0.1 | | | 1.01 | | 8.7 (7.4, 10.2) | | | 0.1 | 1.01 |  |
| Subtype | 1.2 (1.1, 1.4) | | 0.0 | | 1.00 | 6.1 (5.7, 6.5) | | | 0.0 | 1.00 | 6.8 (6.4, 7.3) | | | | 0.0 | 1.00 | | 8.2 (7.6, 8.8) | | 0.0 | | | 1.00 | | 8.6 (7.3, 10.1) | | | 0.0 | 1.00 |  |
| xNMSC | 1.2 (1.1, 1.4) | | 0.0 | | 1.00 | 6.0 (5.6, 6.4) | | | -0.1 | 0.99 | 6.6 (6.1, 7.0) | | | | -0.3 | 0.96 | | 7.6 (7.0, 8.3) | | -0.6 | | | 0.93 | | 8.0 (6.7, 9.6) | | | -0.5 | 0.94 |  |
| First ever | 1.2 (1.1, 1.4) | | 0.0 | | 1.00 | 6.0 (5.6, 6.4) | | | -0.1 | 0.99 | 6.6 (6.1, 7.0) | | | | -0.3 | 0.96 | | 7.6 (7.0, 8.3) | | -0.6 | | | 0.93 | | 8.0 (6.6, 9.6) | | | -0.6 | 0.93 |  |
| C81 **Hodgkin lymphoma** | | |  | |  |  | | |  |  |  | | | |  |  | |  | |  | | |  | |  | | |  |  |  |
| Aggr | 1.8 (1.6, 2.0) | | 0.0 | | 1.00 | 2.0 (1.8, 2.3) | | | 0.0 | 1.02 | 1.2 (1.1, 1.5) | | | | 0.0 | 1.01 | | 2.1 (1.8, 2.4) | | 0.0 | | | 1.00 | | 2.3 (1.6, 3.1) | | | 0.0 | 1.00 |  |
| Subtype | 1.8 (1.6, 2.0) | | 0.0 | | 1.00 | 2.0 (1.8, 2.2) | | | 0.0 | 1.00 | 1.2 (1.1, 1.4) | | | | 0.0 | 1.00 | | 2.1 (1.8, 2.4) | | 0.0 | | | 1.00 | | 2.3 (1.6, 3.1) | | | 0.0 | 1.00 |  |
| xNMSC | 1.8 (1.6, 2.0) | | 0.0 | | 1.00 | 2.0 (1.8, 2.3) | | | 0.0 | 1.01 | 1.2 (1.0, 1.4) | | | | -0.1 | 0.96 | | 2.0 (1.7, 2.4) | | -0.1 | | | 0.96 | | 1.8 (1.2, 2.7) | | | -0.4 | 0.81 |  |
| First ever | 1.8 (1.6, 2.0) | | 0.0 | | 1.00 | 2.0 (1.8, 2.3) | | | 0.0 | 1.01 | 1.2 (1.0, 1.4) | | | | -0.1 | 0.96 | | 2.0 (1.7, 2.4) | | -0.1 | | | 0.96 | | 1.8 (1.2, 2.7) | | | -0.5 | 0.80 |  |
| C82-C86 & C96 **Non-Hodgkin lymphoma** | | | | | | | | |  |  |  | | | |  |  | |  | |  | | |  | |  | | |  |  |  |
| Aggr | 0.9 (0.8, 1.1) | | 0.0 | | 1.02 | 3.4 (3.1, 3.7) | | | 0.0 | 1.01 | 16.2 (15.5, 16.9) | | | | 0.1 | 1.00 | | 50.4 (48.9, 52.0) | | 0.2 | | | 1.00 | | 53.2 (49.8, 56.8) | | | 0.1 | 1.00 |  |
| Subtype | 0.9 (0.8, 1.1) | | 0.0 | | 1.00 | 3.3 (3.0, 3.7) | | | 0.0 | 1.00 | 16.2 (15.5, 16.9) | | | | 0.0 | 1.00 | | 50.2 (48.6, 51.7) | | 0.0 | | | 1.00 | | 53.1 (49.7, 56.7) | | | 0.0 | 1.00 |  |
| xNMSC | 0.9 (0.8, 1.1) | | 0.0 | | 0.99 | 3.3 (3.0, 3.6) | | | 0.0 | 0.99 | 15.6 (14.9, 16.3) | | | | -0.6 | 0.96 | | 48.8 (47.2, 50.5) | | -1.3 | | | 0.97 | | 51.9 (48.3, 55.8) | | | -1.2 | 0.98 |  |
| First ever | 0.9 (0.8, 1.1) | | 0.0 | | 0.99 | 3.3 (3.0, 3.6) | | | 0.0 | 0.99 | 15.5 (14.8, 16.3) | | | | -0.6 | 0.96 | | 48.6 (47.0, 50.2) | | -1.6 | | | 0.97 | | 51.3 (47.7, 55.2) | | | -1.8 | 0.97 |  |
| C88 & C90 **Multiple myeloma** | | | | |  |  | | |  |  |  | | | |  |  | |  | |  | | |  | |  | | |  |  |  |
| Aggr | 0.0 (0.0, 0.0) | |  | |  | 0.4 (0.3, 0.5) | | | 0.0 | 1.03 | 5.6 (5.2, 6.1) | | | | 0.0 | 1.01 | | 22.6 (21.6, 23.7) | | 0.0 | | | 1.00 | | 19.2 (17.2, 21.4) | | | -0.1 | 0.99 |  |
| Subtype | 0.0 (0.0, 0.0) | |  | |  | 0.4 (0.3, 0.5) | | | 0.0 | 1.00 | 5.6 (5.2, 6.0) | | | | 0.0 | 1.00 | | 22.6 (21.5, 23.6) | | 0.0 | | | 1.00 | | 19.4 (17.4, 21.6) | | | 0.0 | 1.00 |  |
| xNMSC | 0.0 (0.0, 0.0) | |  | |  | 0.4 (0.3, 0.5) | | | 0.0 | 0.99 | 5.5 (5.1, 5.9) | | | | -0.1 | 0.98 | | 22.4 (21.3, 23.5) | | -0.2 | | | 0.99 | | 19.3 (17.1, 21.7) | | | -0.1 | 1.00 |  |
| First ever | 0.0 (0.0, 0.0) | |  | |  | 0.4 (0.3, 0.5) | | | 0.0 | 0.99 | 5.5 (5.1, 5.9) | | | | -0.1 | 0.98 | | 22.3 (21.2, 23.4) | | -0.3 | | | 0.99 | | 19.3 (17.1, 21.8) | | | 0.0 | 1.00 |  |
| C91-C95 **Leukemia** | | | | |  |  | | |  |  |  | | | |  |  | |  | |  | | |  | |  | | |  |  |  |
| Aggr | 3.5 (3.3, 3.9) | | 0.1 | | 1.03 | 2.7 (2.4, 3.0) | | | 0.0 | 1.00 | 10.9 (10.3, 11.5) | | | | 0.0 | 1.00 | | 35.1 (33.8, 36.4) | | 0.2 | | | 1.01 | | 35.8 (33.1, 38.8) | | | 0.3 | 1.01 |  |
| Subtype | 3.5 (3.2, 3.8) | | 0.0 | | 1.00 | 2.7 (2.4, 3.0) | | | 0.0 | 1.00 | 10.9 (10.3, 11.4) | | | | 0.0 | 1.00 | | 34.9 (33.6, 36.2) | | 0.0 | | | 1.00 | | 35.6 (32.8, 38.5) | | | 0.0 | 1.00 |  |
| xNMSC | 3.4 (3.2, 3.8) | | 0.0 | | 1.00 | 2.7 (2.4, 3.0) | | | 0.0 | 0.98 | 10.4 (9.9, 11.0) | | | | -0.4 | 0.96 | | 32.6 (31.3, 33.9) | | -2.3 | | | 0.93 | | 34.1 (31.2, 37.3) | | | -1.4 | 0.96 |  |
| First ever | 3.4 (3.2, 3.8) | | 0.0 | | 1.00 | 2.7 (2.4, 3.0) | | | 0.0 | 0.98 | 10.4 (9.9, 11.0) | | | | -0.4 | 0.96 | | 32.3 (31.0, 33.7) | | -2.5 | | | 0.93 | | 34.5 (31.5, 37.7) | | | -1.1 | 0.97 |  |
